# Environmental conditions and marine heatwaves influence blue whale foraging and reproductive effort

**DOI:** 10.1002/ece3.9770

**Published:** 2023-02-26

**Authors:** Dawn R. Barlow, Holger Klinck, Dimitri Ponirakis, Trevor A. Branch, Leigh G. Torres

**Affiliations:** ^1^ Geospatial Ecology of Marine Megafauna Lab, Department of Fisheries, Wildlife, and Conservation Sciences, Marine Mammal Institute Oregon State University Newport Oregon USA; ^2^ K. Lisa Yang Center for Conservation Bioacoustics Cornell University Ithaca New York USA; ^3^ Department of Fisheries, Wildlife, and Conservation Sciences, Marine Mammal Institute Oregon State University Newport Oregon USA; ^4^ School of Aquatic and Fisheries Sciences University of Washington Seattle Washington USA

**Keywords:** acoustics, behavior, blue whale, boosted regression tree models, life history, marine heatwave

## Abstract

Animal behavior is motivated by the fundamental need to feed and reproduce, and these behaviors can be inferred from spatiotemporal variations in biological signals such as vocalizations. Yet, linking foraging and reproductive effort to environmental drivers can be challenging for wide‐ranging predator species. Blue whales are acoustically active marine predators that produce two distinct vocalizations: song and D calls. We examined environmental correlates of these vocalizations using continuous recordings from five hydrophones in the South Taranaki Bight region of Aotearoa New Zealand to investigate call behavior relative to ocean conditions and infer life history patterns. D calls were strongly correlated with oceanographic drivers of upwelling in spring and summer, indicating associations with foraging effort. In contrast, song displayed a highly seasonal pattern with peak intensity in fall, which aligned with the timing of conception inferred from whaling records. Finally, during a marine heatwave, reduced foraging (inferred from D calls) was followed by lower reproductive effort (inferred from song intensity).

## Graphical abstract

Our work links animal behavior and oceanography to provide insights into species life history and investigate the impacts of environmental change. We examine environmental correlates of blue whale vocalization patterns in Aotearoa New Zealand. Our results demonstrate the vulnerability of marine predator populations to marine heatwaves due to the links between environmental factors that control prey availability, foraging effort, and reproductive effort.

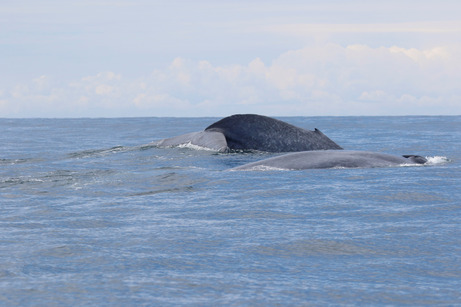

## 1 INTRODUCTION

Variable environmental conditions create a patchy distribution of food resources that animals must effectively navigate for survival (MacArthur & Pianka, 1966). The distribution and behavior of animals therefore reflects fluctuations in their environment and the availability of their prey. If an animal is unable to obtain adequate energy stores through foraging, their ability to successfully produce offspring will also be diminished, potentially impacting population viability (Hirshfield & Tinkle, 1975; McNamara & Houston, 1986). The marine environment is especially dynamic, and mobile predators must therefore respond to shifting prey availability across spatial and temporal scales (Hyrenbach et al., 2000). These mobile marine animals are now faced with additional challenges as rising temperatures due to global climate change are impacting marine ecosystems and organisms worldwide (Hazen et al., 2013; Hoegh‐Guldberg & Bruno, 2010; Poloczanska et al., 2013; Silber et al., 2017; Sydeman et al., 2015). As global ocean temperatures increase, the frequency and intensity of anomalous warm water events known as marine heatwaves are on the rise as well (Frölicher et al., 2018; Oliver et al., 2018). Marine heatwaves can cause a range of ecological consequences, including changes in water column structure, primary production, species composition, marine life distribution and health, and fisheries management such as closures and quota changes (Oliver et al., 2018). The effects of these environmental changes on marine species' ability to successfully feed and reproduce therefore have critical consequences for their survival.

The distribution and habitat use patterns of marine predators often reflect dynamic ecological processes by integrating multiple trophic levels, and therefore their response to changing ocean conditions is tightly linked to shifts in their prey (Cotte et al., 2011; Croll et al., 1998; Nicol et al., 2000; Silber et al., 2017). Marine heatwaves can reduce foraging success in marine predators, which can in turn decrease reproduction and population health. During the North Pacific marine heatwave in 2013–2016, common murres died in record numbers and many breeding colonies of this fish‐eating seabird experienced complete reproductive failure, presumably due to an ecosystem‐wide scarcity of high‐quality forage species (Piatt et al., 2020).

For far‐ranging marine predators such as whales, measuring such changes is challenging. Yet a handful of recent studies have correlated decreased reproductive output with reduced foraging, by either measuring or modeling population demographic parameters. Recent diminishing prey resources appear to be insufficient to support reproduction for North Atlantic right whales, likely contributing to low calving rates (Gavrilchuk et al., 2021). Southern right whale reproductive success is correlated with global climate indices and the density of their prey (Seyboth et al., 2016). Reduced calving rates of gray whales are associated with decreased sea ice cover on their Arctic foraging grounds (Perryman et al., 2021). Blue whales in the North Pacific faced with reduced foraging opportunities due to changing environmental conditions are predicted to suffer population‐level consequences in terms of reproductive success (Pirotta et al., 2019).

While evidence exists that foraging and reproductive success are inextricably linked in marine predators including baleen whales, examining relationships between environmental variation and reproductive output in terms of calf counts in subsequent years misses a key step: the more immediate influence of foraging success on subsequent reproductive effort within the same year. Monitoring biological signals can be an effective tool for overcoming this hurdle, particularly for sparsely distributed, wide‐ranging species for which consistent observation across their range is difficult. Indeed, animals across taxonomic groups send and receive key information via biological signals (Dall et al., 2005), including social, alarm, or food‐associated signals (Clay et al., 2012; Torney et al., 2011). In the marine environment where light attenuates quickly, low‐frequency sound propagates efficiently over long distances (Au & Hastings, 2008). Therefore, many marine species including baleen whales rely on sound as a primary sensory modality, and communication via acoustic signaling is central to behaviors related to foraging and reproduction (Tyack & Clark, 2000).

Blue whales (*Balaenoptera musculus*) are a globally distributed, vocally active species. Here we focus on the pygmy blue whale (*B. m. brevicauda*) population that utilizes the South Taranaki Bight (STB) region between the North and South Islands of Aotearoa New Zealand (Barlow et al., 2018; Torres, 2013). This population is genetically distinct, with an estimated population size of 718 individuals (95% CI = 279–1926; Barlow et al., 2018). Unlike other blue whale populations with stereotypical migrations between low latitude breeding grounds and higher latitude feeding grounds, New Zealand blue whales rely on the same habitat in the STB throughout the year for multiple critical life history processes, including foraging, nursing and raising calves, and potentially breeding (Barlow et al., 2018, 2022). Therefore, the STB region is an ideal study system for year‐round monitoring of multiple life history processes in a single location, uniquely enabling us to address challenging questions about the biology, ecology, and life history of a large marine vertebrate.

The STB region is home to a productive coastal upwelling system, whereby a plume of cold water originating off the northwest of the South Island (Shirtcliffe et al., 1990) supports enhanced primary productivity (Chiswell et al., 2017) and dense aggregations of krill (*Nyctiphanes australis*; Bradford & Chapman, 1988; Bradford‐Grieve et al., 1993) that sustain an important blue whale foraging ground (Barlow et al., 2020, 2021). While blue whale habitat use and distribution patterns have been well described for the STB region during spring and summer months (Barlow et al., 2020, 2021; Barlow & Torres, 2021; Torres et al., 2020), the annual and seasonal patterns of blue whale occurrence in the area remain undescribed. The year‐round presence of upwelling (Chiswell et al., 2017) indicates that blue whales may be able to forage in the STB in all seasons. The region was impacted by well‐documented, severe regional marine heatwave conditions in the summers of 2016 and 2018, with anomalously high temperatures leading to reduced primary productivity and ecosystem‐scale consequences (Chiswell & Sutton, 2020). During the summer 2016 marine heatwave, krill aggregation density was dramatically reduced compared to more typical upwelling conditions, the distribution of krill aggregations shifted further offshore, and blue whale distribution was similarly shifted (Barlow et al., 2020). However, the impact of these marine heatwaves on blue whale foraging and reproductive effort remains unknown.

Blue whales produce two main call types, each with different temporal occurrence patterns and hypothesized biological function. Song is composed of a limited number of sounds that are repeated to form a recognizable pattern (McDonald et al., 2006). These songs are presumed to be signals produced exclusively by males, used to mediate social interactions and maintain associations, and likely play a role in reproduction (Lewis et al., 2018; McDonald et al., 2006; Oleson, Calambokidis, et al., 2007). Across the global oceans, the occurrence patterns of blue whale songs vary seasonally (Barlow et al., 2022; Burtenshaw et al., 2004; Leroy et al., 2018, 2021; Letsheleha et al., 2022; McCauley et al., 2018; Samaran et al., 2013; Stafford et al., 2001; Szesciorka et al., 2020). However, these songs are stereotyped and relatively stable, with characteristics that are distinct between acoustic populations (Leroy et al., 2021; McDonald et al., 2006). The second type of calls are downswept vocalizations known as D calls, which are produced by all sexes and age classes (Lewis et al., 2018; Oleson, Calambokidis, et al., 2007) and common across populations. The function of D calls is less precisely understood, but they are a hypothesized social call, often produced in conjunction with foraging behavior (Cade et al., 2021; Lewis et al., 2018; Oleson, Calambokidis, et al., 2007). Further support for D calls as a signal of foraging comes from their relationship with upwelling‐driven productivity that supports krill availability, the primary prey of blue whales (Barlow et al., 2021; Cade et al., 2021; Szesciorka et al., 2020). Studying the spatial and temporal patterns in calling relative to environmental conditions can foster insight into blue whale ecology and important life history functions (Cade et al., 2021; Oestreich et al., 2022; Szesciorka et al., 2020).

In this study, we analyze the annual cycles and environmental correlates of both blue whale call types detected in continuous recordings in the STB region to examine call function and gain insight into underlying life history patterns (i.e., timing of foraging and reproductive effort). We anticipate different seasonal patterns in song and D calls, with song showing a clear seasonal cycle in intensity that is less correlated with environmental conditions and more temporally stable. We expect D calls to occur more often in the spring and summer during periods of enhanced productivity that support prey aggregations. If D calls indeed indicate foraging effort, we expect lower D call activity during marine heatwaves when upwelling is reduced. Finally, we predict a positive correlation between the number of D call detections and subsequent song intensity, under the premise that more foraging enables more reproductive effort. Therefore, we anticipate that song will be reduced following marine heatwave events when blue whales were not able to obtain adequate energetic stores from feeding. In summary, we examine acoustic signals of blue whales in the STB to describe year‐round occurrence and behavioral patterns, assess correlations with variable ocean conditions, explore relationships between foraging and reproductive effort, and ultimately shed light on potential consequences of climate change for the viability of blue whale populations.

## 2 METHODS

### 2.1 Detection and classification of blue whale calls

**FIGURE 1:**
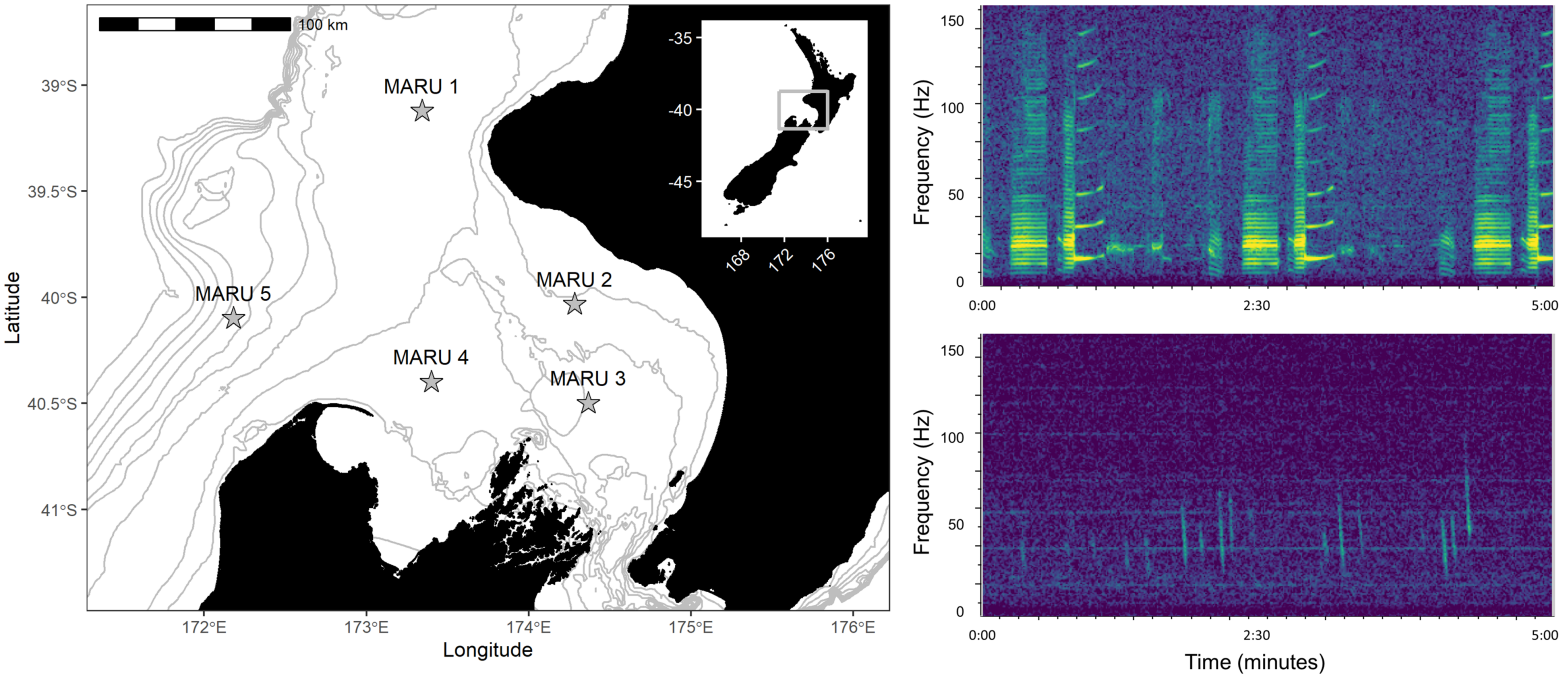
Study area map and blue whale call spectrograms. Left panel: map of the study area in the South Taranaki Bight region, with hydrophone (marine autonomous recording unit; MARU) locations denoted by the stars. Gray lines show bathymetry contours at 50 m depth increments, from 0 to 500 m. Location of the study area within New Zealand is indicated by the inset map. Right panels: example spectrograms of the two blue whale call types examined: the New Zealand song recorded on 31 May 2016 (top) and D calls recorded 20 September 2016 (bottom). Spectrograms are configured with a 3072 point fast Fourier transform, Hann window, 50% overlap.

**FIGURE 2:**
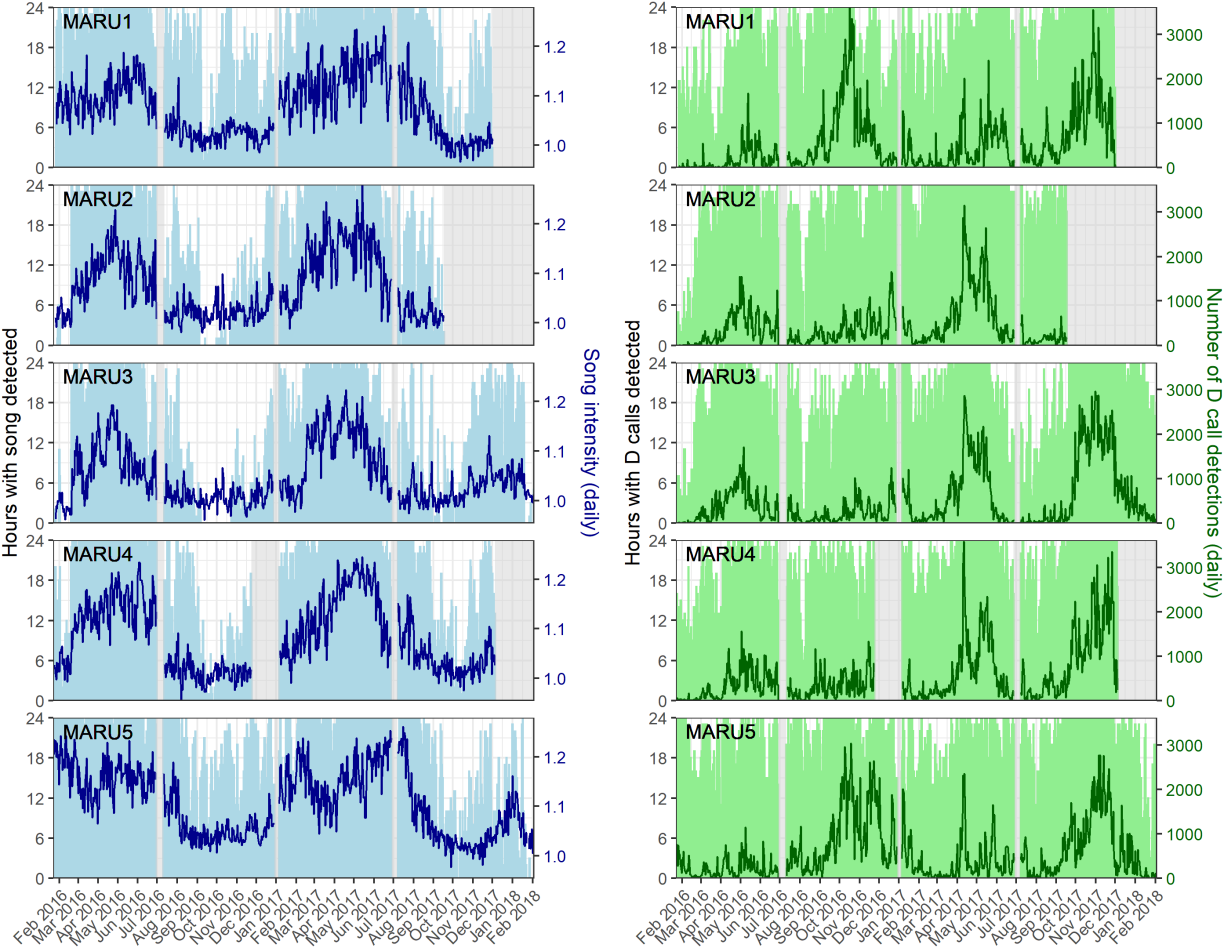
Occurrence patterns of song and D calls. Left panels: number of hours per day with song detected (light blue bars) and daily song intensity index (dark blue lines) for each hydrophone (MARU 1–5) over the entire recording period. Right panels: number of hours per day with D calls detected (light green bars) and number of D call detections per day (dark green lines) for each hydrophone over the entire recording period. The hydrophone is listed at the top of each panel, corresponding to map locations in Figure 1. Periods with light gray shading indicate gaps in recording due to hydrophone refurbishment.

Acoustic data were recorded using five marine autonomous recording units (MARUs; Calupca et al., 2000) deployed in the STB region at depths ranging from 66 to 278 m (Figure 1). The hydrophones had a flat frequency response (±2 dB) in the 15–585 Hz frequency band, a total sensitivity of −145.5 dB, and recorded continuously at a 2 kHz sampling rate with a high‐pass filter at 10 Hz and a low‐pass filter at 800 Hz. Acoustic data were collected continuously from 23 January 2016 to 3 February 2018, with brief gaps in recording approximately every six months for data retrieval and hydrophone refurbishment (Figure 2).

Blue whale vocalizations were identified based on their unique spectral characteristics (Figure 1). All recordings were analyzed using Raven Pro, versions 1.6 and 2.0 (Center for Conservation Bioacoustics, 2019). Spectrogram template correlation detectors (Mellinger & Clark, 2000) were implemented to automatically identify putative instances of the New Zealand song and D calls in the recordings. Five templates were selected for song, and 13 templates were used for D calls to adequately capture the greater variability among calls. Putative call detections were compared against the template with the highest spectrogram correlation score, and detection thresholds of 0.75 for song and 0.80 for D calls were applied after testing a range of possible threshold values. Detector performance was evaluated by comparison to a subset of 26 days, representing one randomly selected day per month across the full recording period. This ground‐truth data set was reviewed separately for each call type, and vocalizations were manually annotated. For song, recordings were reviewed in consecutive 15‐min spectrograms within the 0–50 Hz frequency bandwidth (3000 sample Hann window; 50% overlap). For D calls, recordings were reviewed in 5‐min spectrograms in the 0–150 Hz bandwidth (2048 sample Hann Window, 50% overlap). Automatic detections were considered true positives if they overlapped with a manually annotated call in the ground‐truth data set by at least 50% in time and frequency. Three evaluation metrics were calculated using custom MATLAB scripts: *precision* represents the proportion of detections that were true positives, *recall* is the proportion of true calls that were detected, and the *false alarm rate* is computed as the number of false positives per hour (Mellinger et al., 2016).

After running the song detector on the full data set, detection events were manually reviewed by an analyst at an hourly resolution, ensuring that false positives were not included in the analysis. In addition to the hourly presence or absence of the New Zealand song, a separate metric was computed to measure the intensity of song activity. The song intensity index is calculated as the ratio of the energy in the call frequency bandwidth (23–24 Hz) relative to the energy in selected background frequencies (11, 39 Hz), and a similar approach has been effectively used to describe behavior and phenology of blue whales from changes in this acoustic signal (Oestreich et al., 2020; Širović et al., 2009). Song intensity index was summarized on a daily scale for further analysis. To illustrate the annual cycle of song in the region, the mean song intensity index per day of the year was taken across all five hydrophone locations and the full recording period.

The spectrogram template correlation detector for D calls prioritized minimizing missed detections, which meant many false positives were retained by the detector and needed to be removed from the data set prior to further analysis. We therefore created an automatic classification algorithm to classify putative D call detections as either true D calls or false positives. Putative D call detections for the period between January 2016 and March 2017 were manually reviewed, and false positives were identified. Subsequently, a suite of spectral measurements (Appendix A) was extracted in Raven for all call detections identified by the detector and manually classified as true D calls or false positives and used as a training library to develop the automatic classification algorithm. A random forest model was fitted to the data using the “ranger” package in R (Wright & Ziegler, 2017), with the classification as either true D calls or false positives as the response variable and the spectral features extracted in Raven as the predictors. The number of trees used to grow the random forest model (ntree) was set to 100, and the number of variables sampled as candidates at each split (mtry) was set to 7. The training library was used to develop and evaluate whether the random forest model could reliably be used for automated classification by training the random forest model on a subset of 75% of the data and predicting to the withheld 25%. The misclassification rate, false‐negative rate, true‐positive rate, false‐positive rate, and true‐negative rate were calculated (Table A1); this process was repeated over 100 bootstrap iterations, and the mean and standard deviation were calculated for the evaluation metrics across all bootstrap runs. To assess whether using the automatic classifier would impact ecological inference from blue whale calling patterns, temporal occurrence patterns were compared between calls identified via the manual validation and automated classification methods for the period between January 2016 and March 2017. This comparison was evaluated visually and using Pearson's correlation coefficient. Subsequently, the automatic classifier was run on the full data set, and the number of hours per day with D calls present and total D calls per day were computed for the entire recording period. To illustrate the annual cycle of D calls in the region, the mean D call detections per day of the year were taken across all five hydrophone locations and the full recording period.

### 2.2 Call detection range estimation

Sound propagation models were generated to estimate the listening range of each hydrophone unit using the range‐dependent acoustic model (RAM; Collins, 1993). RAM is well suited for calculating detection areas in low‐frequency soundscapes and shallow water environments like the STB and does so by simulating call propagation from a whale to a hydrophone under the conditions at the time. The model incorporates numerous soundscape features that impact the detection range for calling blue whales, including sound speed profile through the water column, depth of the hydrophone receiver, seafloor substrate characteristics, depth of the calling whale, the source level and frequency of the vocalizations of interest, and ambient noise.

Sound speed profiles were based on the World Ocean Atlas (WOA2009), bathymetry was extracted at a 1‐arc minute resolution from the ETOPO1 data set (Amante & Eakins, 2009), and geo‐acoustic parameters for fine sand (Bostock et al., 2019; Wentworth, 1922; grain size phi = 3) were used in the propagation model. The depth of the calling whale was set to 25 m. The source levels and dominant frequency band differ between song and D calls, and therefore can influence detection area. While source levels have not empirically been estimated for blue whales in New Zealand, we obtained these parameters from the literature for application in the models. For song, the source level of 179 dB re 1 μPa at 1 m estimated for pygmy blue whales from the Australian population was used as a proxy (Gavrilov et al., 2011), and the dominant frequency band used was 17–50 Hz. For D calls, the source level was set to 174 dB re 1 μPa at 1 m (Samaran et al., 2010), and the frequency band was 20–100 Hz. Hourly ambient noise levels were considered the 1st percentile levels of the dominant frequency band for each call type. Calls were simulated across a grid of points at 1‐arc minute resolution (~2.25 km^2^) at varying distances surrounding each hydrophone, and detection area was estimated given the ambient noise recorded at the hydrophone. To summarize the modeled detection distances, we calculated the mean daily detection area for each call type at each hydrophone across the entire recording period.

### 2.3 Environmental data

Environmental variables were chosen based on prior investigations into the oceanographic properties of regional upwelling processes and functional drivers of blue whale foraging opportunities in the STB (Barlow et al., 2020, 2021; Barlow & Torres, 2021). Daily SST and SST anomaly measurements were acquired from the multiscale ultrahigh resolution (MUR) satellite at a 0.01‐degree spatial resolution and daily temporal scale. Net primary productivity of carbon (NPP) was obtained from the Moderate Resolution Imaging Spectroradiometer (MODIS) satellite at a 0.0125‐degree spatial resolution and 8‐day composite to minimize the impact of cloud cover. All satellite data were accessed via the ERDDAP server (https://coastwatch.pfeg.noaa.gov/erddap) using the R package “rerddapXtracto” (Mendelssohn, 2019). For each day with acoustic recordings, SST, SST anomaly, and NPP were extracted within the calculated detection radius surrounding each hydrophone, separately for song and D calls. The mean values were then computed within the detection area to summarize the environmental conditions within the possible range of detection for vocalizing blue whales over the full data set. NPP measurements were log‐transformed for all subsequent analyses to minimize skew.

### 2.4 Whaling data

Whaling records were accessed from the International Whaling Commission individual catch database (Allison, 2020). Extracted data (where available) included lengths of fetuses from pregnant blue whales that were killed, along with the date and location of the catch. Based on length frequencies at different positions, data were separated between Antarctic blue whales (*B. m. intermedia*) and pygmy blue whales (*B. m. brevicauda*). Catches north of the following latitudes and south of the equator were assumed to be pygmy blue whales: 20–30° E (46°S), 30–70°E (52°S), 70–80°E (53°S), and 80°E–180° (52°S). All catches longer than 24.2 m were assigned to Antarctic blue whales and catches south of this pygmy region but specifically recorded as “pygmy” were assigned to pygmy blue whales. Catches in the Atlantic Ocean (west of 20°E) were classified as Antarctic, while those off the west coast of South America were excluded as they belong to the Chilean blue whale population (Branch et al., 2021; Figure A6).

For Antarctic blue whales, a wide variety of recording methods were used by different nations, and most lengths were recorded in feet and rounded to the nearest foot. For pygmy blue whales, nearly all pygmy blue whale catches were taken either by Japan (1959/60–1963/64) or the Soviet Union (1962/63–1971/72), and lengths were recorded in metric units, mostly to the nearest cm. Note that for the Soviet data, information has been reconstructed from original logbooks for nearly all expeditions and replaces the original falsified records that were submitted to the International Whaling Commission from 1949/50 (Yablokov, 1994; Zemsky et al., 1995).

The pygmy data in the New Zealand region (145°E–180°, 32–50°S) were assessed to determine whether these blue whales followed similar seasonal patterns of fetal growth as other blue whales in the Southern Hemisphere. All catches in this region come from Soviet expeditions and were caught while the Soviet fleets were returning from the Antarctic during February–May in the years 1964–1968 and 1973. The Soviet fleets in the database are listed under the following codes: 6300 (*Slava*), 6442 and 6445 (both *Sovetskaya Ukraina*), and 6490 (*Sovetskaya Rossia*).

The fetal length measurements throughout the year were then used to infer the timing of births, based on the time at which fetal length measurements reach their maximum. Subsequently, timing of births was used to infer timing of conception, based on prior analyses of the catch records that concluded a gestation period of about 11 months for Antarctic blue whales (Laws, 1959; Mackintosh & Wheeler, 1929).

### 2.5 Statistical analyses

We constructed models to examine the functional relationships between calling activity and environmental correlates, for both song and D calls across the full recording period. Boosted regression trees (BRT) are a machine learning multivariate modeling approach that combines decision tree methods with a boosting algorithm that iteratively optimizes model performance by combining a large number of decision trees (Elith et al., 2008). This approach is well suited for predicting distribution patterns, describing nonlinear relationships and can handle complex interactions between predictors. Furthermore, BRTs minimize the effect of temporal autocorrelation by randomly selecting a proportion of the data (called the bag fraction) at each iteration, and data are further split during cross‐validation to evaluate model performance. Therefore, the probability of a contiguous timeseries being maintained is quite small. Models were fitted separately for each of the five hydrophones, for two reasons: first, the detection areas between hydrophones occasionally overlap which could lead to overrepresentation of call activity if combined, and second, a main objective was to examine how relationships between calling and environmental drivers varies between locations, therefore combining all hydrophones into a single model would mask any spatial heterogeneity in the functional relationships among hydrophone locations.

All BRT models were implemented using the “gbm” (Greenwell et al., 2019) and “dismo” (Hijmans et al., 2017) packages in R. For song, the response variable is the daily song intensity index, fit with a Gaussian distribution. For D calls, the response variable is number of D calls per day, fit with a Poisson distribution, which is suitable for count data. Full models were first fit for each call type across all seasons at each hydrophone, and subsequently models were fit for within‐season peaks for each call type, at each hydrophone. Dynamic predictor variables included SST, SST anomaly, and log(NPP). Additionally, month was included to examine seasonal effects, and detection area was included to account for variation in the distance over which calls could be detected. All models were fit with a learning rate of 0.005, a bag fraction of 0.75, and a tree complexity of 2. Performance was assessed with two metrics, the cross validated percent deviance explained (cv.dev) and the cross‐validated correlation (cv.cor). The influence of predictor variables on calling activity was assessed in two ways, through the percent contribution in the BRT model and using partial dependence plots to visualize each functional relationship between predictor and response while all other predictors are held at their mean value, implemented using the “pdp” package in R (Greenwell, 2017).

The summers of 2016 and 2018 were characterized by well‐documented marine heatwaves leading to dramatically increased temperatures and reduced primary productivity throughout the STB region, whereas summer 2017 consisted of more typical upwelling conditions (Barlow et al., 2020; Chiswell & Sutton, 2020). Therefore, key upwelling‐related metrics and D calls were compared for just the summer months (January–February) between the three years. Linear mixed models were used to estimate the effect of year while accounting for differences between hydrophone locations, with separate models for each of the three metrics of interest as the response variable: SST, NPP, and D call detections. Subsequently, post hoc tests with a Tukey correction were run to examine pairwise comparisons between years for each metric.

Finally, we fit a linear mixed model to examine the relationship between D call activity during the summer and song intensity in the subsequent fall, accounting for differences between hydrophone locations as a random factor. Calling was averaged by hydrophone, for each season in each year. This analysis covered two years, the marine heatwave year 2016 and the more typical upwelling year in 2017.

## 3 RESULTS

### 3.1 Call detection and classification

**FIGURE A1:**
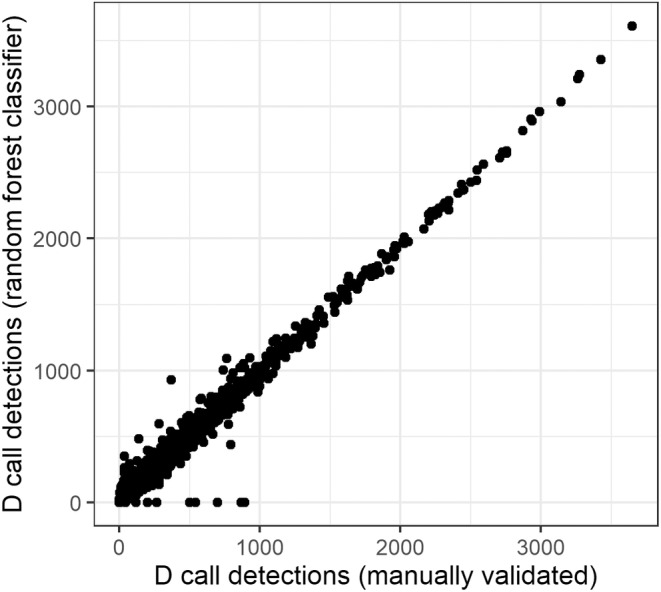
Correlation between the daily number of D calls identified from the manual validation vs. D calls identified by the automatic classifier, for the period 23 January 2016–31 March 2017.

**FIGURE A2:**
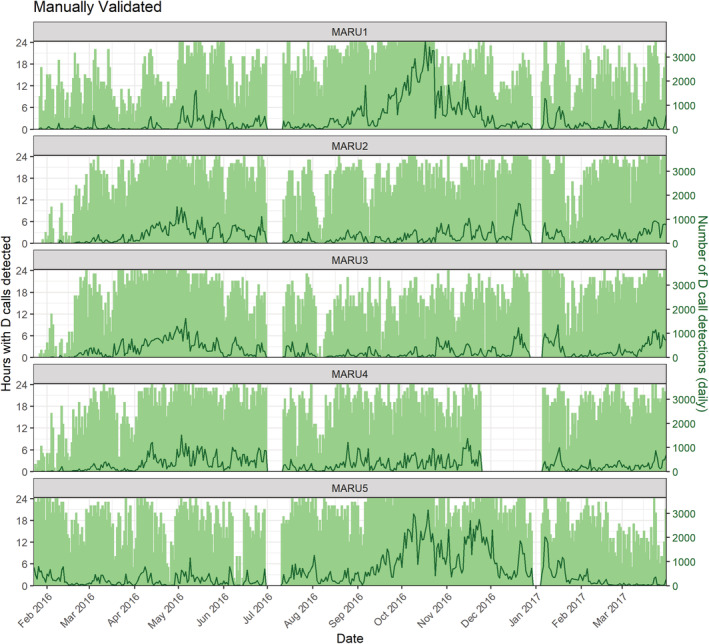
Spatiotemporal detection patterns of blue whale D call occurrence and intensity visualized using D call detections that were manually validated. Hours with D calls detected per day (light green bars) and the daily number of D call detections (dark green lines) are shown between 23 January 2016 and 31 March 2016, at each hydrophone recording location.

**FIGURE A3:**
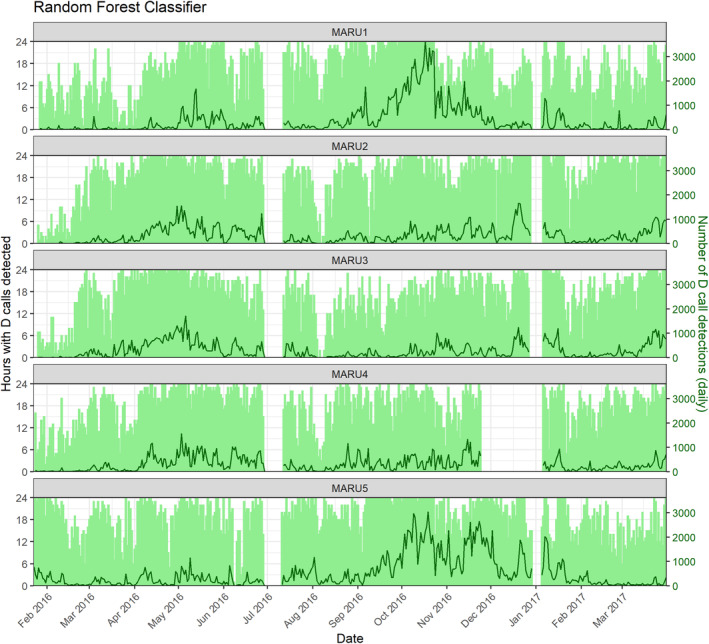
Spatiotemporal detection patterns of blue whale D call occurrence and intensity visualized using D call detections that were automatically classified using the random forest model. Hours with D calls detected per day (light green bars) and the daily number of D call detections (dark green lines) are shown between 23 January 2016 and 31 March 2016, at each hydrophone recording location. Note no noticeable difference in occurrence patterns from Figure A2.

Spectrogram correlation detectors implemented for automatic call detection showed higher performance for the more stereotyped song than for the more variable D call vocalizations. The detectors yielded a precision score of 0.81 for song and 0.51 for D calls, a recall score of 0.96 for song and 0.81 for D calls, and a false alarm rate of 8.18 false positives per hour for song and 13.22 false positives per hour for D calls. For D calls, the detector identified 2.57 million putative calls in the full recording data set. Manual validation of 1.33 million detection events during the period of January 23, 2016–March 31, 2017 contained 51.5% true D calls and 49.5% false positives. The automatic classifier calibrated using this manual validation data set successfully minimized false negatives and false positives while maximizing true negatives and true positives from the putative D call detections (Table A1). Cross‐correlation analysis revealed a highly significant relationship between the number of D calls from the manual validation and automatic classifier (Pearson's correlation coefficient = .991, *p* < 2.2 × 10^−16^; Figure A1). The temporal occurrence patterns visualized using the D calls identified by the automatic classifier were nearly indistinguishable from the patterns revealed by the manually validated calls (Figures A2 and A3). These comparisons indicate that the fully automated methods for detection and classification of D calls provide a robust approach to examine blue whale occurrence patterns and ecological drivers of calling behavior (Figure 2).

### 3.2 Spatial and temporal calling patterns

Both blue whale song and D calls were present across the entire recording period, with blue whale vocalizations detected nearly every single day (Figure 2). While the hourly presence of both song and D calls reveals year‐round presence in the region, the seasonal patterns in the intensity of calling throughout the year differ by call type. Song intensity generally peaked between April and June, with slightly more song activity at the two offshore hydrophone locations (MARU1 and MARU5) where the peak in song intensity begins earlier and extends later than at the hydrophones within the STB (MARU2, MARU3, and MARU4). The period between September and December represents the lowest song intensity across all hydrophones, although song is still present on most days during that time (Figure 2).

D calls occurred in high numbers throughout the year, with high hourly presence and over 3000 calls detected per day at multiple hydrophones in some instances (Figure 2). The offshore hydrophones (MARU1 and MARU5) showed a peak in D calls in the spring through early summer, between October 2016 and January 2017. A similar springtime peak was evident across all five hydrophones in 2017–2018. A secondary fall peak in detections occurred between April and May across all five hydrophones but was stronger in 2017 than in 2016. While song showed a more cyclical pattern in intensity throughout the year, D call detections showed multiple large and small peaks within each year.

**FIGURE 3:**
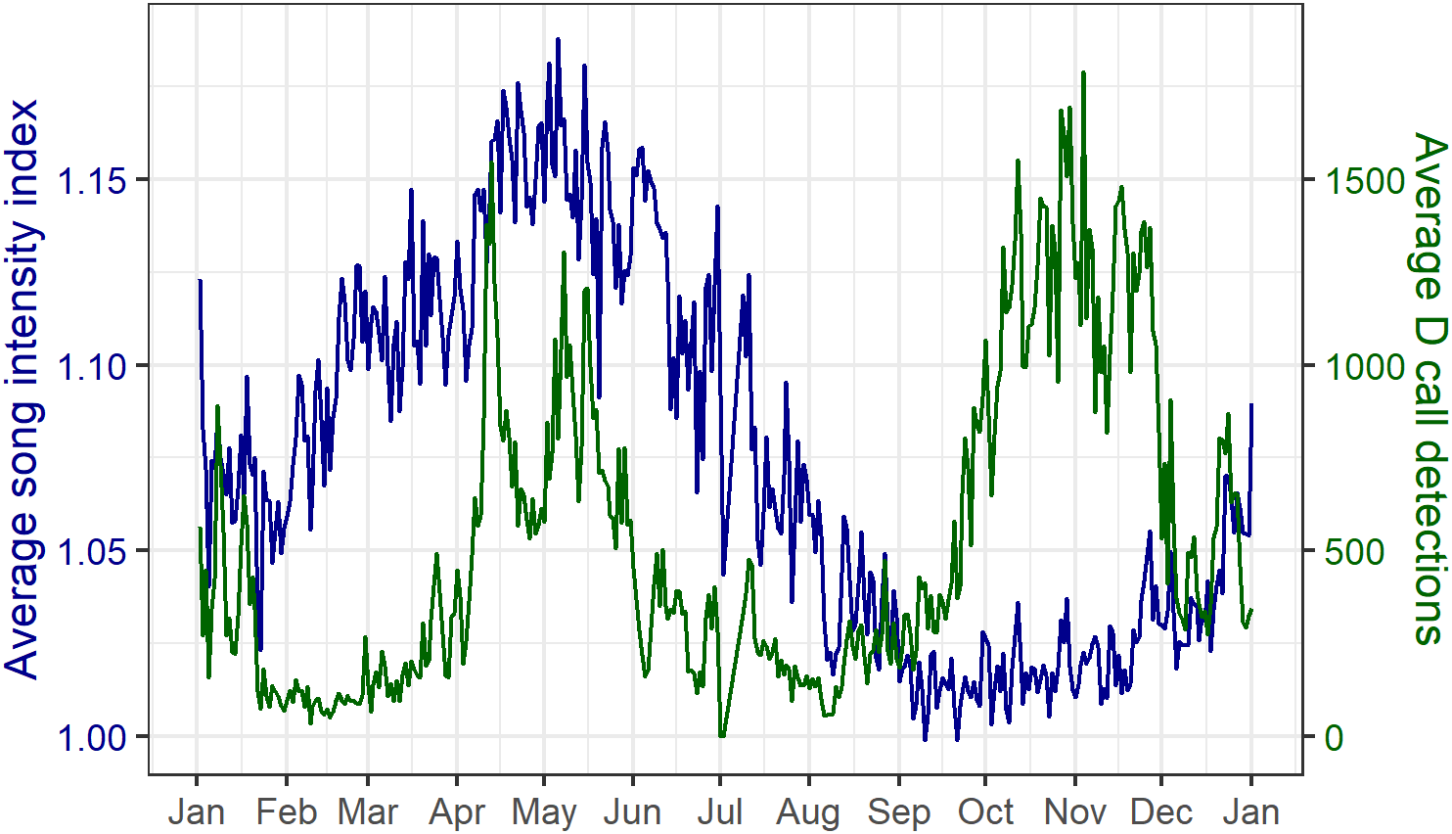
Annual cycle of calling activity. Average annual cycle in the song intensity index (dark blue) and D calls per day of the year, computed across all hydrophone locations and the entire recording period.

The average annual cycle of blue whale vocalizations revealed three seasonal peaks in calling activity, representing both concordance and divergence between the two call types (Figure 3). Song has a single annual peak in the fall, between April and June. D calls have a strong spring peak, between October and January. Subsequently, D calls have a second peak in fall that overlaps with the fall song peak, though in a narrower temporal window between April and May. These periods of elevated calling activity were examined in the within‐peak models.

### 3.3 Environmental correlates of calling

**FIGURE 4:**
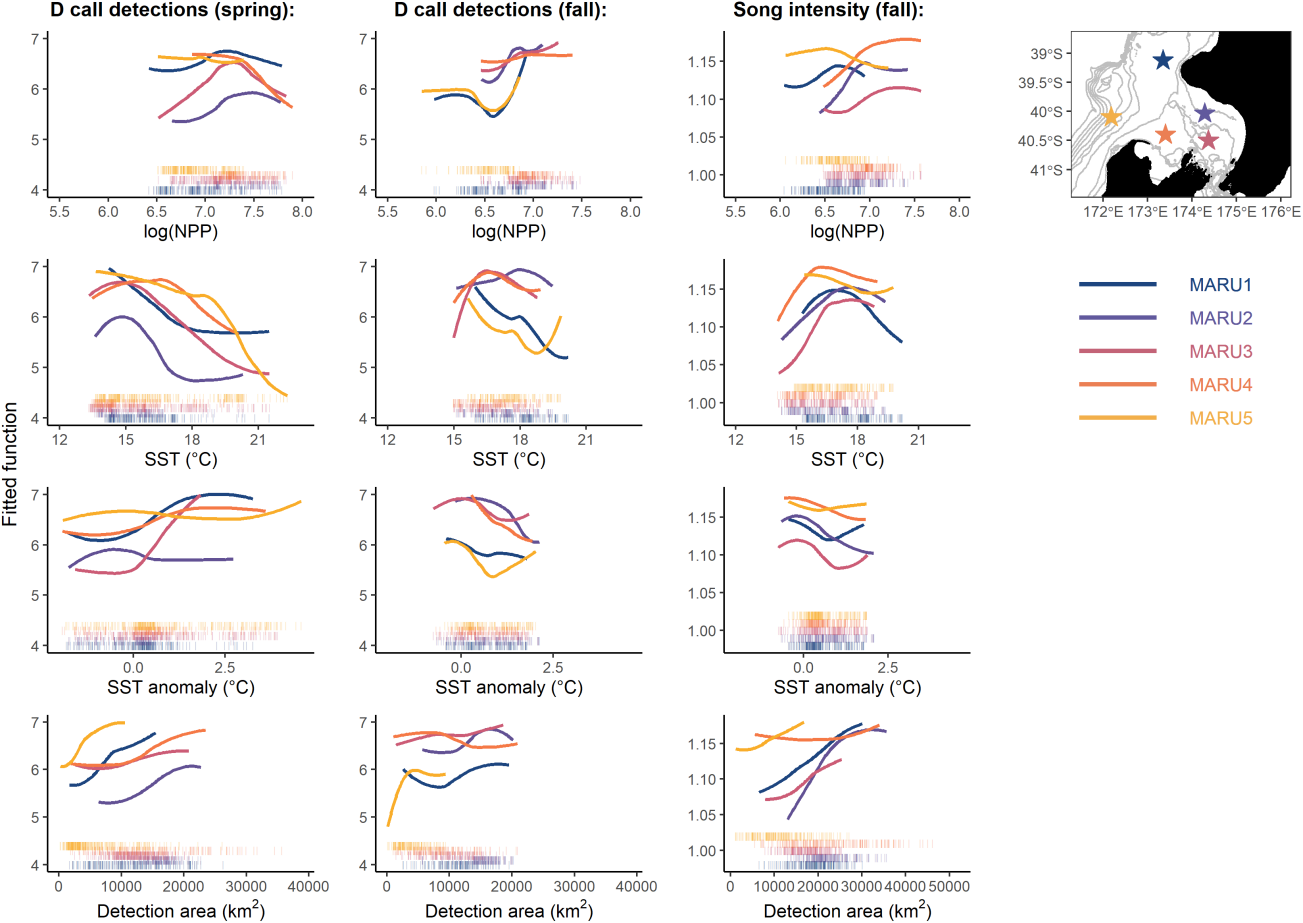
Functional response curves for environmental correlates of calling activity. Partial dependence plots derived from the boosted regression tree fitted for each within‐season peak in calls, showing the smoothed functional relationships between calling (either song intensity index or D call detections) and each predictor variable while fixing all other variables at their mean value. Color is indicative of the hydrophone, with locations shown on the map in upper right panel. Rug plots show distribution of the values for each predictor.

Boosted regression tree (BRT) models performed well overall, with some variability among models fit to different call types, seasons, and hydrophones (Table 1). The models with the greatest performance measured by cross‐validated percent deviance explained (cv.dev) and cross‐validated correlation (cv.cor) were those fit to the full recording period, for song and D calls. Within‐peak models were fitted for each period with the highest calling activity of each call type and showed more nuanced differences among environmental drivers at different times of the year (Table 2, Figure 4). Across all within‐peak models for each hydrophone location, model performance dropped with the removal of month as a predictor (Table 1), but the patterns revealed by functional relationships within the different peaks yield insight into the importance of different environmental correlates seasonally.

**TABLE 1:** Boosted regression tree model performance.

Hydrophone	n trees	cv.Dev	cv.Cor
Song (full)			
MARU1	3300	0.794	0.893
MARU2	5850	0.852	0.923
MARU3	5900	0.789	0.885
MARU4	3050	0.831	0.914
MARU5	4650	0.779	0.883
D calls (full)			
MARU1	6200	0.708	0.837
MARU2	4450	0.701	0.827
MARU3	6100	0.733	0.847
MARU4	4300	0.666	0.807
MARU5	5500	0.697	0.850
D calls (spring)			
MARU1	3750	0.726	0.850
MARU2	2600	0.236	0.467
MARU3	4050	0.708	0.836
MARU4	2150	0.605	0.832
MARU5	4100	0.673	0.809
D calls (fall)			
MARU1	1000	0.466	0.630
MARU2	1800	0.623	0.806
MARU3	2700	0.509	0.734
MARU4	1250	0.363	0.572
MARU5	2150	0.359	0.634
Song (fall)			
MARU1	2750	0.522	0.733
MARU2	2550	0.679	0.803
MARU3	2350	0.679	0.822
MARU4	2150	0.481	0.672
MARU5	2850	0.266	0.537

*Note*: Evaluation of the boosted regression tree models fitted for each call type and hydrophone location. All models were fit with a learning rate of 0.005, a bag fraction of 0.75, and a tree complexity of 2. For song, the response variable is the daily song intensity index, fit with a Gaussian distribution. For D calls, the response variable is number of D calls per day, fit with a Poisson distribution, which is suitable for count data. Full models were first fit for each call type across all seasons at each hydrophone, and subsequently models were fit for within‐season peaks for each call type, at each hydrophone. Performance is assessed with two metrics, the cross validated percent deviance explained (cv.dev) and the cross‐validated correlation (cv.cor).

**TABLE 2:** Boosted regression tree model predictor variable contributions.

Predictor	MARU1	MARU2	MARU3	MARU4	MARU5
Song (full)					
logNPP	11.24	8.50	11.28	6.39	8.19
Month	66.63	59.62	58.08	77.28	70.78
SST	6.16	13.59	15.73	9.20	7.79
SST anomaly	4.13	6.68	7.38	3.04	8.41
Detection area	11.53	11.60	7.50	4.07	4.81
D calls (full)					
logNPP	46.39	40.35	21.18	12.74	11.37
Month	33.82	45.78	47.55	44.83	64.26
SST	3.55	5.11	9.69	9.76	5.76
SST anomaly	3.75	5.71	15.42	13.69	7.23
Detection area	12.46	3.02	6.13	18.95	11.36
D calls (spring)					
logNPP	38.26	85.15	18.67	11.29	16.87
SST	13.43	5.42	35.78	26.21	44.08
SST anomaly	8.98	4.74	33.58	21.13	7.60
Detection area	39.31	4.67	11.95	41.35	31.43
D calls (fall)					
logNPP	48.34	37.66	37.53	16.88	25.94
SST	29.05	11.94	29.12	22.69	26.92
SST anomaly	13.90	38.71	21.05	36.34	32.68
Detection area	8.69	11.66	12.28	24.07	14.44
Song (fall)					
logNPP	24.10	39.11	29.79	29.73	73.00
SST	25.92	15.50	44.58	44.44	11.48
SST anomaly	19.78	15.13	13.14	13.83	5.11
Detection area	30.18	30.24	12.47	11.98	10.39

*Note*: Variable contribution for the boosted regression tree models for song and D calls at each of the hydrophones. The percent contribution in the model is listed for each predictor. The full models with all seasons included are reported first for both call types, followed by the within‐season peaks for each call type.

**FIGURE A4:**
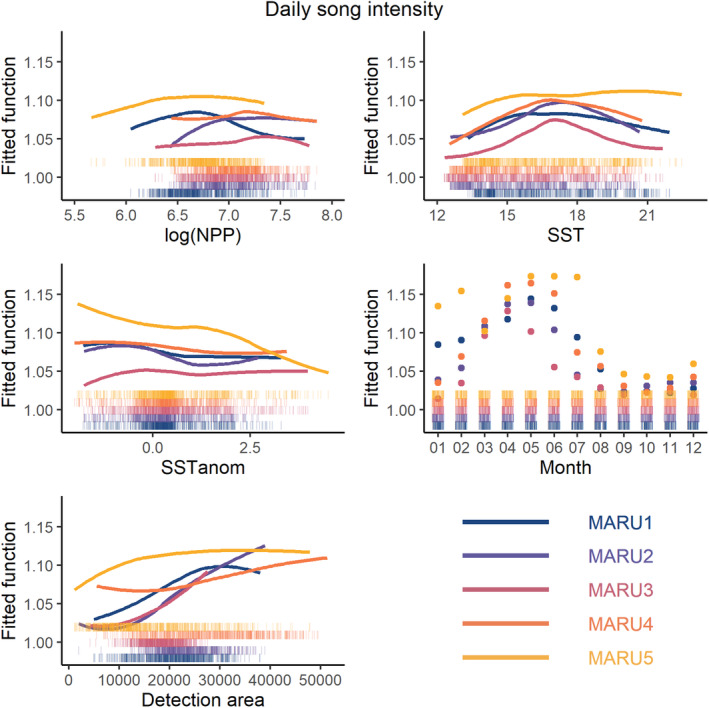
Partial dependence plots derived from the boosted regression tree models for song, showing the smoothed functional relationships between song intensity and each predictor variable while fixing all other variables at their mean value. Color is indicative of the hydrophone. Rug plots show distribution of the values for each predictor, and color corresponds to the hydrophone.

Song intensity showed the strongest relationship with month, which contributed 59%–77% of the explained deviance in the full models (Table 2). As was revealed by the seasonal calling plots (Figure 3), the functional relationship between song intensity and month in the BRT models shows a peak in song intensity between April–June (Figure A4). In contrast, all environmental predictors had relatively low contributions in the full models for song (Table 2). Within the fall peak, song intensity corresponds to an optimal SST range of 16–18°C, lower SST anomalies, and shows an inconsistent relationship with log(NPP) between the hydrophones (Figure 4). There was an increase in song intensity with increased song detection area across all hydrophones.

**FIGURE A5:**
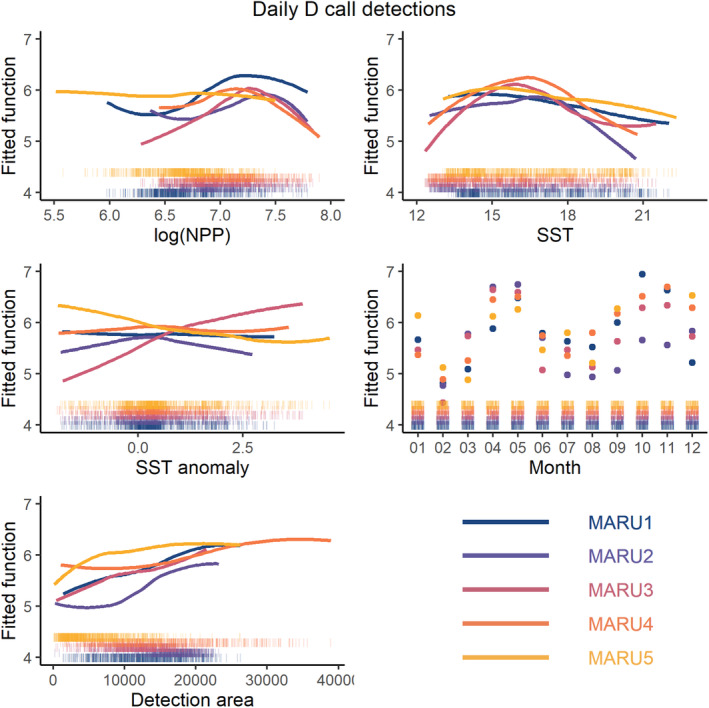
Partial dependence plots derived from the boosted regression tree models for D calls, showing the smoothed functional relationships between D call detections and each predictor variable while fixing all other variables at their mean value. Color is indicative of the hydrophone. Rug plots show distribution of the values for each predictor, and color corresponds to the hydrophone.

Environmental correlates had stronger contribution to the full models for D calls than song (Table 2). Month still had a strong influence in the full models, with D call detections at their highest in the spring and fall. The number of D call detections increased with the detection area for all hydrophones (Table 2; Figure A5). Model performance was higher for D calls in spring than in fall, and functional relationships differed between the two seasons (Table 1, Figure 4). In spring, increased D calls are associated with lower SST, positive SST anomalies, and increase with increasing log(NPP) before leveling off or even decreasing at very high values. In the fall, increased D calls are associated with higher log(NPP) values and lower SST anomalies. Furthermore, functional relationships differed more strongly between hydrophone locations in fall than in spring. For example, D calls in the fall showed a negative relationship with increasing SST at the offshore hydrophones (MARU1 and MARU5), whereas MARU2, MARU3, and MARU4 showed an optimal SST range of 16–18°C, much like what was observed for the fall peak in song. Overall, the functional response curves for D calls in the fall more closely resemble the functional relationships for song in fall than they do D calls in spring (Figure 4).

### 3.4 Call function and life history patterns

**FIGURE 5:**
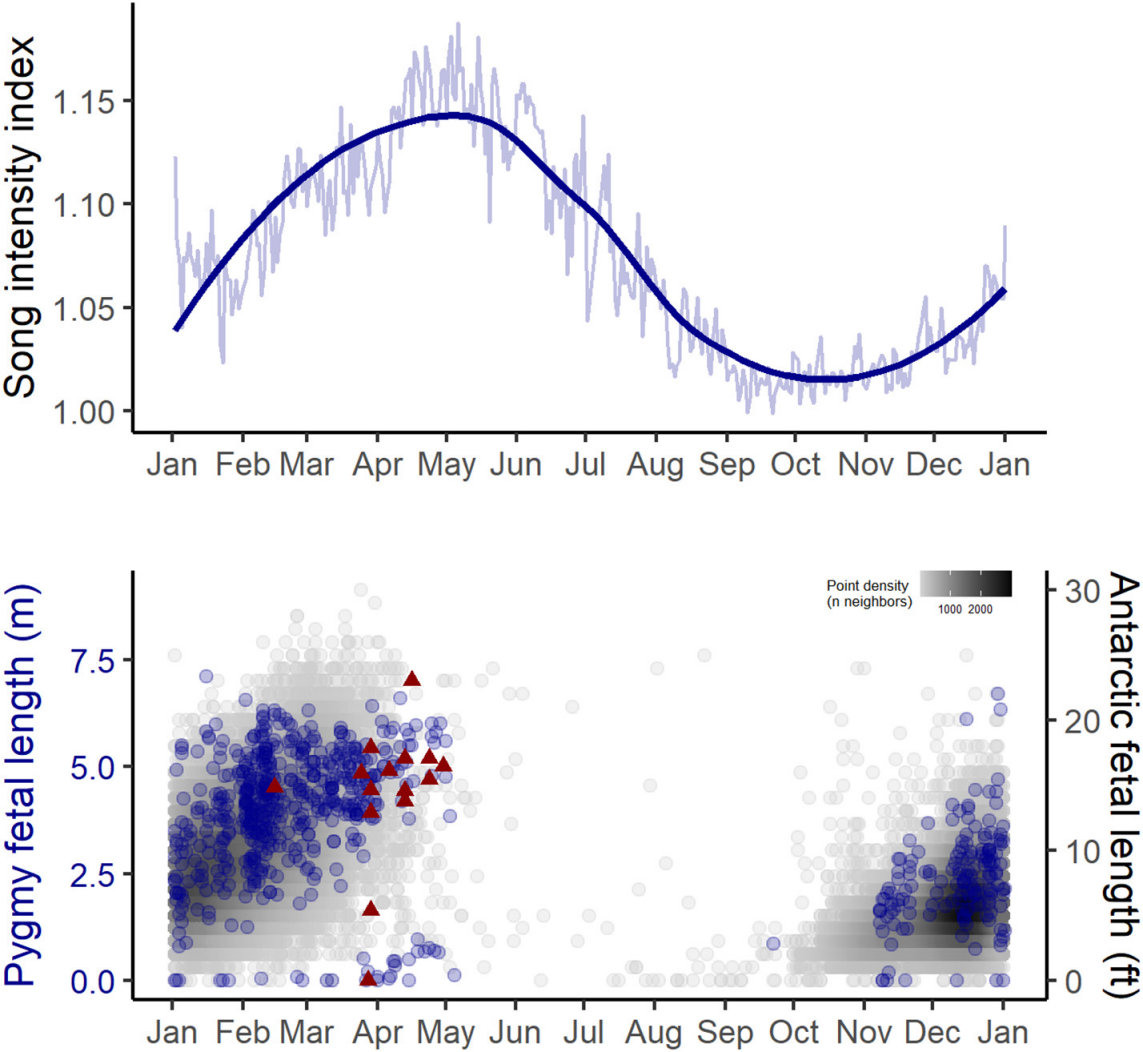
Annual song intensity and the breeding cycle. Top panel: average yearly cycle in song intensity index, computed across the five hydrophone locations and the entire recording period; dark blue line represents a loess smoothed fit. Bottom panel: fetal length measurements from whaling catch records for Antarctic blue whales (gray, measurements rounded to the nearest foot), pygmy blue whales in the southern hemisphere (blue, measurements rounded to the nearest centimeter). Measurements from blue whales caught within the established range of the New Zealand population are denoted by the dark red triangles. Calving presumably takes place around or shortly after fetal lengths are at their maximum (April–May), which implies that mating likely occurs around May–June, coincident with the peak song intensity. *Note*: Many small fetuses were missed in the Antarctic blue whale data, whereas sampling was more thorough for pygmy blue whale data.

Song intensity was at its highest between April July, with the peak taking place in May–June (Figure 5). Examination of whaling catch records from the New Zealand region revealed alignment with the overall pattern of fetal length measurements for pygmy and Antarctic blue whales caught in the Southern Hemisphere (Figure 5). Calving presumably takes place around, or shortly after, the time when fetal lengths are at their maximum, indicating that births occur in April–May. Based on this timing, we infer that mating likely happens in May–June (assuming an 11‐month gestation [Laws, 1959; Mackintosh & Wheeler, 1929]), which coincides with the peak song intensity. Therefore, the mean peak in recorded song intensity aligns closely with the estimated timing of conception.

**FIGURE 6:**
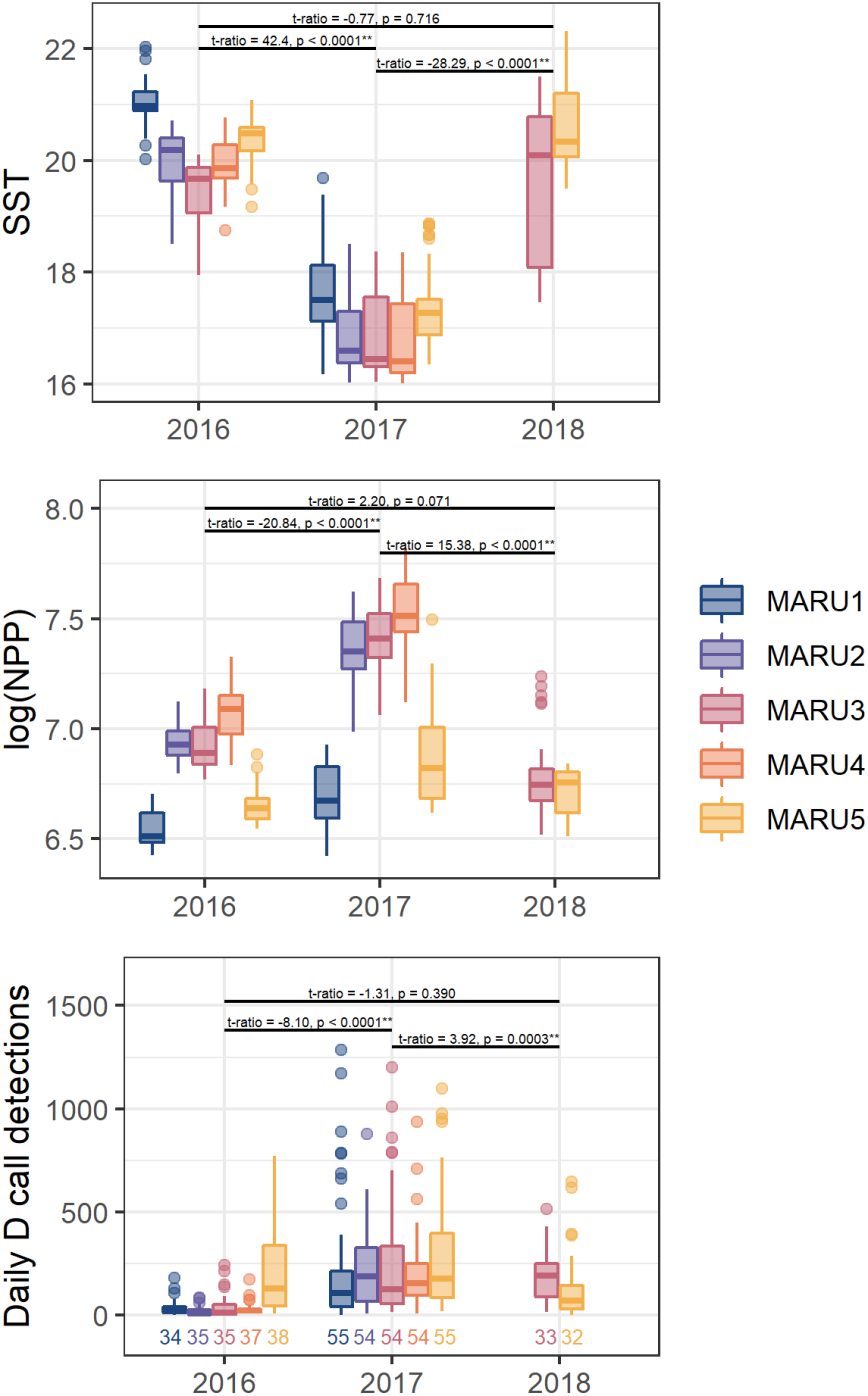
Environmental conditions and D calls in summer months. Mean sea surface temperature (top), net primary productivity (middle), and daily D call detections (bottom) for the period of 1 January through 28 February in each of the three years with recording coverage, with each of the five hydrophone recording locations indicated by colors. The number of recording days for each hydrophone in each year are indicated in the bottom panel. January and February were characterized by regional marine heatwaves in 2016 and 2018, while 2017 exhibited more typical summer upwelling conditions. Pairwise comparisons resulting from a linear mixed model for each variable by year (accounting for differences among hydrophone locations) are indicated above each set of boxplots. In all cases, the linear mixed models were significant, with the two marine heatwave years characterized by comparable SST, log(NPP), and D call values that were significantly different from those measured in the more typical upwelling year.

In 2016 and 2018 when documented marine heatwaves took place (Chiswell & Sutton, 2020), SST was higher, log(NPP) was lower, and mean daily D call detections were lower within the detection range surrounding each of the hydrophone locations (Figure 6). Linear mixed models accounting for differences among hydrophone locations revealed significant interannual differences for SST (*χ* ^2^ = 2131.2, *p* < 2.0 × 10^−16^), NPP (*χ* ^2^ = 3462.7, *p* < 2.0 × 10^−16^), and D calls (*χ* ^*2*^ = 69.6, *p* = 7.6 × 10^−16^). Furthermore, pairwise post hoc comparisons showed significant differences between 2016–2017 and 2017–2018, but not 2016–2018 (Figure 6).

**FIGURE 7:**
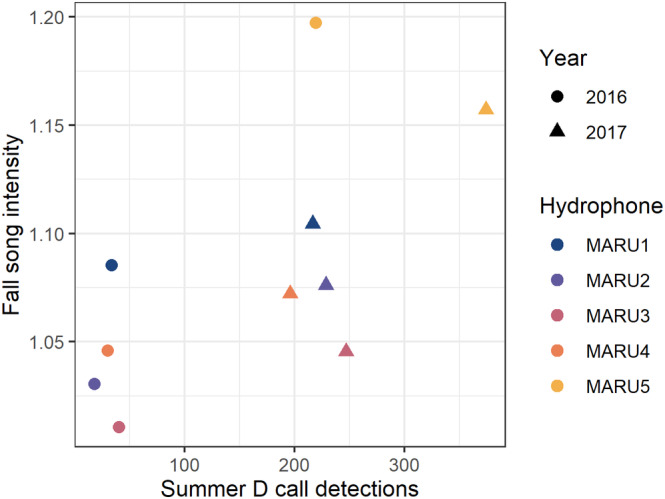
Relationship between summer D call detections and fall song intensity. Relationship between the mean number of D call detections between 1 January through 28 February and the mean song intensity index in the subsequent fall peak between 1 April and 31 June. Points are symbolized by year and hydrophone recording location.

A linear mixed model determined a significant positive relationship between mean daily D call detections in the summer and mean song intensity in the subsequent fall peak (*χ* ^2^ = 4.15, *p* = .04; Figure 7). Lower summertime D call activity during the 2016 marine heatwave was followed by reduced song intensity during the inferred breeding period later in the same year, whereas more D call activity in 2017 was followed by higher fall song intensity. The notable exception to this pattern occurs at the MARU5 hydrophone location (Figure 7).

## 4 DISCUSSION

Our analyses of spatial and temporal calling patterns and environmental correlates from two years of acoustic monitoring data demonstrate how ocean conditions that drive prey availability for blue whales influence their D call activity during the primary foraging season, yet song intensity was primarily related to the temporal variable month. These results support the previously hypothesized life history functions of song and D calls for blue whales (Lewis et al., 2018; Oleson, Calambokidis, et al., 2007). Moreover, we link the peak song intensity to the time of mating inferred from fetal growth patterns (Figure 5), demonstrating that New Zealand blue whales likely use the STB as a breeding ground and song intensity reflects their reproductive cycle. D calls were reduced during marine heatwaves when upwelling conditions (i.e., low SST, high NPP) were reduced or absent (Figures 4 and 6). These periods also corresponded with a documented reduction in both the number of krill aggregations and krill aggregation density in the region, indicating poor blue whale foraging conditions (Barlow et al., 2020). Interestingly, D calls during the summertime are correlated with song intensity during the following fall reproductive peak (Figure 7), with lower D call rates followed by lower song intensity. Thus, our findings suggest a negative impact of marine heatwave conditions on multiple life history functions at the population scale (foraging and breeding effort) and demonstrate how climate change may impact blue whale populations through reduced feeding opportunities, with potential consequences for reproduction and population viability.

### 4.1 Ecological patterns of calling

Using prior research on physical and biological drivers of blue whale foraging opportunities in the region (Barlow et al., 2020, 2021; Barlow & Torres, 2021; Torres et al., 2020), models examining calling activity as a function of season and environmental predictors performed well (Table 1) and illustrated how different environmental features play different roles throughout the year. For example, the performance of the D call within‐peak models is higher in the spring than in the fall, indicating that the relationships between environmental drivers and D call activity are more pronounced in the spring and early summer during the predominant upwelling season (Barlow et al., 2021; Chiswell et al., 2017). Notably, environmental correlates of D calls in the fall bear more similarities to song during the fall than to D calls in the spring (Figure 4). For example, in the springtime, D calls show a strong negative relationship with SST. In the fall, both song and D calls display a mid‐range SST optimum between 16 and 18°C (Figure 4). This finding may be a function of different available environmental conditions during these periods, with different drivers of foraging conditions than indicated by prior studies conducted in the spring and summer (Barlow et al., 2020, 2021; Barlow & Torres, 2021), or a different function of D calls between the spring and fall peaks. The double‐peak pattern of D calls (Figure 3) is also observed in the California Current system (Lewis et al., 2018; Oleson, Wiggins, & Hildebrand, 2007) and in the Corcovado Gulf of Chile (Buchan et al., 2021). In all three regions, it seems the fall peak in D calls could represent late‐season foraging associated with a fall bloom in productivity, a transition into more social behaviors relating to reproductive activity, or both.

While the environmental correlates of the spring D call peak are strongly indicative of upwelling and conditions suitable for blue whale foraging (Barlow et al., 2020, 2021; Barlow & Torres, 2021), the driving patterns behind the fall calling peaks are less evident from environmental correlates alone. Song is hypothesized to serve a reproductive function (McDonald et al., 2006; Oleson, Calambokidis, et al., 2007), and while mating has never been directly observed for blue whales, the paradigm for baleen whales is a temporally defined breeding season each year. Therefore, the phenology of the fall peak in song may be driven more predominantly by the breeding season defined by their life history timing rather than environmental cues. While the hypothesized foraging function of D calls aligns closely with our observations during the spring and summer, the fall peak in D calls aligns more closely with song, both in terms of timing and environmental correlates (Figures 3 and 4). Although song is only produced by males, other demographic groups engage in social activities, and D calls are produced by all sexes and age classes (Lewis et al., 2018; Oleson, Calambokidis, et al., 2007). The coincident fall peak of both call types may represent a period of social behavior across demographic groups during the likely breeding season for this blue whale population. This potential use of D calls as social calls more broadly is corroborated by increased D call detections coincident with blue whale super‐aggregations (Cade et al., 2021) and triads engaged in social behaviors potentially related to reproductive activity (Schall et al., 2019).

By including detection area in the models, we accounted for the potential of increased call detections with larger detection ranges while evaluating the ecological impacts of environmental predictor variables. Model results revealed that the role of environmental features differs among hydrophone locations in the STB. The presence and strength of the upwelling plume presents a distinct temperature signal closer to the source, where cold water is brought to the surface near the MARU5 location (Barlow et al., 2021; Shirtcliffe et al., 1990). Further downstream along the plume's trajectory, there is a stronger signal in surface productivity (Chiswell et al., 2017). This pattern is evident in the model results for D calls in spring, illustrated by the high contribution of SST and low contribution of NPP for MARU5 compared to low contribution of SST and high contribution of NPP for MARU1 (Table 2). Many patterns are summarized in Figure 4, including nuanced spatial and seasonal differences among functional response curves. For D calls in fall, the functional relationships with SST present an interesting spatial split between inshore and offshore locations: the response curves for MARU1 and MARU5 mirror the patterns of D calls in spring, whereas MARU2, MARU3, and MARU4 show the same optimal temperature range as song in fall. This distinction of functional response curves between the offshore and inshore hydrophones is evident for all environmental predictors of D calls in fall (Figure 4, center panels). These spatial patterns in the relationships between calling and oceanographic features reflect the heterogenous patterning of environmental gradients and upwelling dynamics across the STB region.

Due to the inability to localize calling whales and paucity of information on cue rates for blue whales in this population, it was not possible to obtain any estimates of actual whale density in the area. This limitation makes it challenging to separate changes in calling activity from changes in whale density. Indeed, photo‐identification records have shown that blue whales that feed in the STB region during the summers are resighted elsewhere around New Zealand (Barlow et al., 2018), and acoustic recordings indicate occasional presence of the New Zealand song off the coast of eastern Australia and as far north as Tonga (Balcazar et al., 2015; McCauley et al., 2018). However, given the near‐constant presence of blue whale calls in the STB (Figure 2), the differences in occurrence patterns between song and D calls, and near‐equal sex ratios of blue whales throughout their range (Branch & Monnahan, 2021), changes in calling activity likely do reflect underlying patterns in ecology and life history rather than solely changes in distribution or density.

### 4.2 Reproductive cycle inferred from song and whaling records

While seasonal cycles in song activity are evident for blue whale populations around the world (Barlow et al., 2022; Buchan et al., 2020; Lewis et al., 2018; McCauley et al., 2018; Samaran et al., 2013; Stafford et al., 2001, 2004), it is often challenging to disentangle changes in song detection from movement of blue whales into and out of an area. In the STB, blue whales are present year round (Barlow et al., 2022; Warren et al., 2021), minimizing the confounding variable of animals moving out of the area (Figures 2 and 3). The whales forage (Barlow et al., 2020; Torres et al., 2020) in the same area where they sing, indicating they use the same place for multiple critical life history functions (Barlow et al., 2022). While the reproductive function of song has been postulated based on behavioral observations over short time scales (Lewis et al., 2018; Oleson, Calambokidis, et al., 2007), we make the link via the timing of reproduction inferred from biological data on the timing of conception, fetal growth, and births over a broad spatial and temporal scale. Therefore, the cycle of song intensity in this region is likely indicative of the annual breeding cycle for this blue whale population.

**FIGURE A6:**
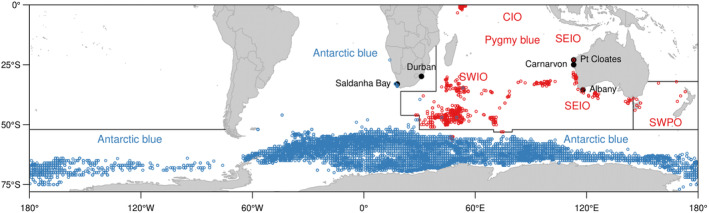
Whaling catches of Antarctic blue whales (blue) and pygmy blue whales (red) in the southern hemisphere, with assumed boundaries in black between each. Acronyms denote each of the currently assumed blue whale populations: central Indian ocean (CIO, Sri Lanka), south‐west Indian Ocean (SWIO, Madagascar), south‐east Indian Ocean (SEIO, Australia/Indonesia), south‐west Pacific Ocean (SWPO, New Zealand). Key land stations in and near the pygmy blue whale region are labeled.

Temporal patterns of blue whale fetal length measurements from whaling records (Allison, 2020) indicate that mating for this population may occur in May–June, after maximum fetal length measurements in April–May that indicate birth. This proposed mating coincides with our recorded peak song intensity, supporting the hypothesis that song is used in reproductive efforts and that blue whales mate during May–June in the STB (Figure 5). While the sample size of blue whale catches with fetal length measurements from the New Zealand region is small (*n* = 15), they reflect the pattern in measurements from all other pygmy blue whales taken in the Southern Hemisphere, and Antarctic blue whale catches, both of which are far more numerous (pygmy *n* = 716, Antarctic *n* = 29,883; Figure 5, Figure A6).

### 4.3 Potential marine heatwave impacts on foraging and reproduction

Reduced upwelling during the 2016 and 2018 marine heatwaves (Chiswell & Sutton, 2020; Oliver et al., 2017; Sutton & Bowen, 2019) coincided with a significant reduction in D calls across all five hydrophone locations (Figure 6). The decrease in D calls during these periods further emphasizes their role as a foraging‐related call in the spring and summer, indicating decreased feeding during anomalously warm periods. The marine heatwave conditions and impacts on blue whale foraging may serve as a harbinger of what is to come, as ocean temperatures are expected to warm and marine heatwaves are expected to increase in both frequency and intensity with climate change (Frölicher et al., 2018; Holbrook et al., 2019; Oliver et al., 2018), including in the STB region (Behrens et al., 2022).

**FIGURE A7:**
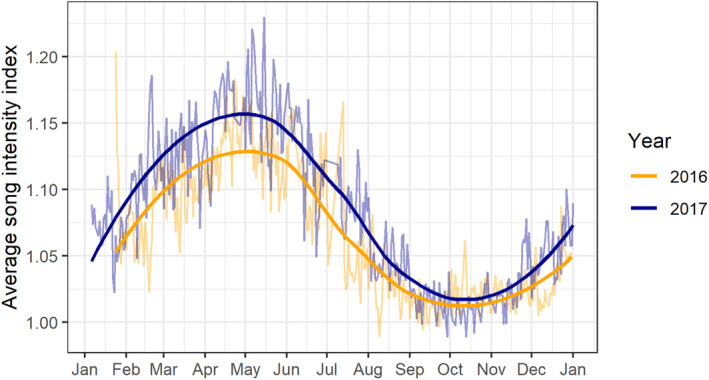
Average annual cycle in song intensity index across all five hydrophone locations by year. Dark solid lines represent a loess smoothed fit. Note the alignment of the fall peak in song intensity in both years, but the lower average song intensity in 2016 than in 2017.

Reduced foraging opportunities may have ramifications for population viability if blue whales are not able to obtain sufficient energetic stores to support reproduction (Pirotta et al., 2019). Furthermore, there are potential carryover effects into subsequent years if enough energy is not obtained in a foraging season (Soledade Lemos et al., 2020). This consequence of reduced foraging was indicated by the significant relationship between summertime D call activity and song intensity in the subsequent breeding season. The timing of the fall peak in song did not differ between 2016 and 2017, but the mean song intensity index was lower during the 2016 fall peak (Figure A7). The reduction in foraging during the 2016 marine heatwave may have led to reduced reproductive activity or animals moving away from this region in the peak breeding period (Figure 7). The clear exception in this pattern is the MARU5 hydrophone, which is the furthest southwest recording location. Visual surveys during the 2016 heatwave found that blue whales were absent from the central STB; sightings were concentrated offshore, where concurrent prey mapping identified the only location with high krill density in summer 2016 (Barlow et al., 2020). During this heatwave, the western region of the STB may have served as a refuge for krill, and blue whales may have altered their distribution in response to shifting prey availability, as reflected in both the sightings data (Barlow et al., 2020) and higher than expected D call detections. The increased song intensity at the furthest offshore hydrophone in 2016 does not follow the expected pattern (Figure 7), perhaps due to concentration of blue whales further offshore following the summer 2016 marine heatwave (Barlow et al., 2020).

The impacts of marine heatwaves are wide reaching. The 2014–2016 marine heatwave in the California Current led to widespread changes in the biological structure and composition of pelagic and coastal ecosystems (Cavole et al., 2016), including anomalously low primary productivity (Kahru et al., 2018), reductions in krill abundance by up to 95% (Lavaniegos et al., 2019), shifts in the timing and location of spawning of pelagic fish stocks (Auth et al., 2018), and compression of humpback whale habitat leading to higher recorded entanglements in fishing gear (Santora et al., 2020). The 2015–2016 Tasman Sea heatwave reduced primary productivity (Chiswell & Sutton, 2020), altered zooplankton community composition (Evans et al., 2020), severely impacted the aquaculture industry (Oliver et al., 2017), and shifted blue whale distribution in the STB region during the summer foraging season (Barlow et al., 2020). While these impacts of marine heatwaves have been documented across ecosystems and regions, the link to reproductive success in baleen whales has remained a gap in knowledge. Environmental fluctuations and reduced foraging are known to impact population health in multiple baleen whale species, including blue (Pirotta et al., 2019), gray (Lemos et al., 2021; Soledade Lemos et al., 2020), and right (Gavrilchuk et al., 2021; Seyboth et al., 2016) whales. While we could not investigate population vital rates with the data at‐hand, the information on blue whale call function gained through this study enabled us to postulate a connection between reduced foraging during marine heatwaves and subsequent reduced reproductive activity by monitoring acoustic signals. Based on regionally resolved model projections, marine heatwaves in the Tasman Sea are expected to increase in intensity and duration, including potentially annually persistent marine heatwave conditions by the end of the century under some greenhouse gas emissions scenarios (Behrens et al., 2022). Therefore, the implications of our findings will become increasingly pertinent for the future of the New Zealand blue whale population.

Our analyses of blue whale vocalizations shed light on call function, associations with variable environmental conditions including reduced foraging during marine heatwaves, and a potential relationship between foraging and reproductive effort. Looking forward, extension of this work over longer time scales to assess the influence of environmental variability on blue whale calling, behavior, and reproduction is prudent, particularly considering the anticipated impacts of climate change and the likelihood of nonanalogous conditions in the future (Frölicher et al., 2018).

## AUTHOR CONTRIBUTIONS

**Dawn R. Barlow:** Conceptualization (equal); formal analysis (lead); investigation (lead); methodology (equal); visualization (lead); writing – original draft (lead). **Holger Klinck:** Investigation (supporting); methodology (equal); supervision (supporting); writing – review and editing (supporting). **Dimitri Ponirakis:** Formal analysis (supporting); investigation (supporting); methodology (equal). **Trevor A. Branch:** Formal analysis (supporting); investigation (supporting); methodology (equal); writing – review and editing (supporting). **Leigh G. Torres:** Conceptualization (lead); funding acquisition (lead); investigation (equal); methodology (equal); project administration (lead); supervision (lead); writing – review and editing (lead).

## CONFLICT OF INTEREST

The authors declare that they have no competing interests.

## Data Availability

The processed data sets generated for this study and relevant analysis code are available via the Figshare Digital Repository: https://doi.org/10.6084/m9.figshare.21836733.v2.

## APPENDIX A

The following features were extracted for all call detections identified by the D call spectrogram correlation template detector and manually classified as true D calls or false positives in Raven:

**Table:**

delta time low frequency (Hz) high frequency (Hz) hour of day aggregate entropy (bits) average entropy (bits) average power density (dB FS) bandwidth 50% (Hz) bandwidth 90% (Hz) center frequency (Hz) center time (s) relative center time delta frequency (Hz) duration 50% (s) duration 90% (s) energy (dB) frequency 5% (Hz) relative frequency 5% frequency 25% (Hz) relative frequency 25%	frequency 75% (Hz) relative frequency 75% frequency 95% (Hz) relative frequency 95% inband power (dB FS) length (frames) max entropy (bits) max frequency (Hz) max time (s) min entropy (bits) peak frequency (Hz) peak frequency contour average slope (dB ms) peak frequency contour max frequency (Hz) peak frequency contour max slope (dB ms) peak frequency contour min frequency (Hz) peak frequency contour min slope (dB ms)	peak frequency contour number of infinite points peak power density (dB FS) peak tie (s) relative peak time SNR (dB) sample length (samples) time 5% (Hz) relative time 5% time 25% (Hz) relative time 25% time 75% (Hz) relative time 75% time 95% (Hz) relative time 95% template ID template detector correlation score

**TABLE A1:** Results of the random forest classifier evaluation, both for training data and when the classifier predicted to test data.

Metric	Calculation	Mean (training)	SD (training)	Mean (test)	SD (test)
Misclassification rate	1 − (TP + TN/total calls)	0.111	0.00023	0.107	0.00052
False negative rate	FN/FN + TP	0.100	0.00026	0.098	0.00063
True positive rate	TP/TP + FN	0.899	0.00026	0.901	0.00063
False positive rate	FP/FP + TN	0.126	0.00041	0.121	0.00095
True negative rate	TN/TN + FP	0.873	0.00041	0.878	0.00095

*Note*: Each bootstrap iteration trained the model on a randomly selected 75% of the data, and predicted to the withheld 25%, and this process was repeated over 100 runs.

## References

[ece39770-bib-0001] Allison, C. (2020). IWC individual catch database Version 7.1 .

[ece39770-bib-0002] Amante, C. , & Eakins, B. W. (2009). ETOPO1 1 Arc‐Minute Global Relief Model: Procedures, Data Sources and Analysis. In *NOAA Technical Memorandum NESDIS NGDC‐24* . https://www.ngdc.noaa.gov/mgg/global/relief/ETOPO1/docs/ETOPO1.pdf

[ece39770-bib-0003] Au, W. W. L. , & Hastings, M. C. (2008). Principles of marine bioacoustics. In Principles of marine bioacoustics. Springer. 10.1007/978-0-387-78365-9

[ece39770-bib-0004] Auth, T. D. , Daly, E. A. , Brodeur, R. D. , & Fisher, J. L. (2018). Phenological and distributional shifts in ichthyoplankton associated with recent warming in the Northeast Pacific Ocean. Global Change Biology, 24, 259–272. 10.1111/gcb.13872 28948709

[ece39770-bib-0005] Balcazar, N. E. , Tripovich, J. S. , Klinck, H. , Nieukirk, S. L. , Mellinger, D. K. , Dziak, R. P. , & Rogers, T. L. (2015). Calls reveal population structure of blue whales across the Southeast Indian Ocean and the Southwest Pacific Ocean. Journal of Mammalogy, 96(6), 1184–1193. 10.1093/jmammal/gyv126 26989263 PMC4794612

[ece39770-bib-0006] Barlow, D. R. , Bernard, K. S. , Escobar‐Flores, P. , Palacios, D. M. , & Torres, L. G. (2020). Links in the trophic chain: Modeling functional relationships between in situ oceanography, krill, and blue whale distribution under different oceanographic regimes. Marine Ecology Progress Series, 642, 207–225. 10.3354/meps13339

[ece39770-bib-0007] Barlow, D. R. , Klinck, H. , Ponirakis, D. , Garvey, C. , & Torres, L. G. (2021). Temporal and spatial lags between wind, coastal upwelling, and blue whale occurrence. Scientific Reports, 11(6915), 1–10. 10.1038/s41598-021-86403-y 33767285 PMC7994810

[ece39770-bib-0008] Barlow, D. R. , Klinck, H. , Ponirakis, D. , Holt Colberg, M. , & Torres, L. G. (2022). Temporal occurrence of three blue whale populations in New Zealand waters from passive acoustic monitoring. Journal of Mammalogy. 10.1093/jmammal/gyac106

[ece39770-bib-0009] Barlow, D. R. , & Torres, L. G. (2021). Planning ahead: Dynamic models forecast blue whale distribution with applications for spatial management. Journal of Applied Ecology, 58(11), 2493–2504. 10.1111/1365-2664.13992

[ece39770-bib-0010] Barlow, D. R. , Torres, L. G. , Hodge, K. B. , Steel, D. , Baker, C. S. , Chandler, T. E. , Bott, N. , Constantine, R. , Double, M. C. , Gill, P. , Glasgow, D. , Hamner, R. M. , Lilley, C. , Ogle, M. , Olson, P. A. , Peters, C. , Stockin, K. A. , Tessaglia‐Hymes, C. T. , & Klinck, H. (2018). Documentation of a New Zealand blue whale population based on multiple lines of evidence. Endangered Species Research, 36, 27–40. 10.3354/esr00891

[ece39770-bib-0011] Behrens, E. , Rickard, G. , Rosier, S. , Williams, J. , Morgenstern, O. , & Stone, D. (2022). Projections of future marine heatwaves for the oceans around New Zealand using New Zealand's earth system model. Frontiers in Climate, 4(798287), 1–13. 10.3389/fclim.2022.798287

[ece39770-bib-0012] Bostock, H. , Jenkins, C. , Mackay, K. , Carter, L. , Nodder, S. , Orpin, A. , Pallentin, A. , & Wysoczanski, R. (2019). Distribution of surficial sediments in the ocean around New Zealand/Aotearoa. Part B: Continental shelf. New Zealand Journal of Geology and Geophysics, 62(1), 24–45. 10.1080/00288306.2018.1523199

[ece39770-bib-0013] Bradford, J. M. , & Chapman, B. (1988). Nyctiphanes australis (euphausiacea) and an upwelling plume in western cook strait, New Zealand. New Zealand Journal of Marine and Freshwater Research, 22(2), 237–247. 10.1080/00288330.1988.9516296

[ece39770-bib-0014] Bradford‐Grieve, J. M. , Murdoch, R. C. , & Chapman, B. E. (1993). Composition of macrozooplankton assemblages associated with the formation and decay of pulses within an upwelling plume in greater cook strait, New Zealand. New Zealand Journal of Marine and Freshwater Research, 27(1), 1–22. 10.1080/00288330.1993.9516541

[ece39770-bib-0015] Branch, T. A. , & Monnahan, C. C. (2021). Sex ratios in blue whales from conception onward: Effects of space, time, and body size. Marine Mammal Science, 37(1), 290–313. 10.1111/mms.12741

[ece39770-bib-0016] Branch, T. A. , Monnahan, C. C. , Širović, A. , Harthi, S. A. , Allison, C. , Balcazar, N. E. , Barlow, D. R. , Calderan, S. , Cerchio, S. , Michael, C. , Dréo, R. , Gavrilov, A. N. , Gedamke, J. , Hodge, K. B. , Jenner, K. C. S. , Leroy, E. C. , McCauley, R. D. , Miksis‐Olds, J. L. , Miller, B. S. … Willson, M. S. (2021). Monthly movements and historical catches of pygmy blue whale populations inferred from song detections. In *International Whaling Commission SC/68C/SH/17* .

[ece39770-bib-0017] Buchan, S. J. , Balcazar‐Cabrera, N. , & Stafford, K. M. (2020). Seasonal acoustic presence of blue, fin, and minke whales off the Juan Fernández archipelago, Chile (2007–2016). Marine Biodiversity, 50(76), 1–10. 10.1007/s12526-020-01087-3

[ece39770-bib-0018] Buchan, S. J. , Iván, P.‐S. , Diego, N. , Leonardo, C. , Stafford, K. M. , Baumgartner Mark, F. , Arnoldo, V. L. , Paulina, M. , Laura, G. , Constanza, R. , Giovanni, D. , & Sergio, N. (2021). Intraseasonal variation in Southeast Pacific blue whale acoustic presence, zooplankton backscatter, and oceanographic variables on a feeding ground in northern Chilean Patagonia. Progress in Oceanography, 199, 102709. 10.1016/j.pocean.2021.102709

[ece39770-bib-0019] Burtenshaw, J. C. , Oleson, E. M. , Hildebrand, J. A. , McDonald, M. A. , Andrew, R. K. , Howe, B. M. , & Mercer, J. A. (2004). Acoustic and satellite remote sensing of blue whale seasonality and habitat in the Northeast Pacific. Deep Sea Research Part II: Topical Studies in Oceanography, 51, 967–986. 10.1016/j.dsr2.2004.06.020

[ece39770-bib-0020] Cade, D. E. , Fahlbusch, J. A. , Oestreich, W. K. , Ryan, J. , Calambokidis, J. , Findlay, K. P. , Friedlaender, A. , Hazen, E. , Seakamela, S. M. , & Goldbogen, J. A. (2021). Social exploitation of extensive, ephemeral, environmentally controlled prey patches by supergroups of rorqual whales. Animal Behaviour, 182, 251–266. 10.1016/j.anbehav.2021.09.013

[ece39770-bib-0021] Calupca, T. A. , Fristrup, K. M. , & Clark, C. W. (2000). A compact digital recording system for autonomous bioacoustic monitoring. Journal of the Acoustical Society of America, 108, 2582. 10.1121/1.4743595

[ece39770-bib-0022] Cavole, L. M. , Demko, A. M. , Diner, R. E. , Giddings, A. , Koester, I. , Pagniello, C. M. L. S. , Paulsen, M.‐L. , Ramirez‐Valdez, A. , Schwenck, S. M. , Yen, N. K. , Zill, M. E. , & Franks, P. J. S. (2016). Biological impacts of the 2013–2015 warm‐water anomaly in the Northeast Pacific: Winners, losers, and the future. Oceanography, 29(2), 273–285. 10.5670/oceanog.2016.32

[ece39770-bib-0023] Center for Conservation Bioacoustics . (2019). Raven Pro: Interactive Sound Analysis Software . http://www.birds.cornell.edu/raven

[ece39770-bib-0024] Chiswell, S. M. , & Sutton, P. J. H. (2020). Relationships between long‐term ocean warming, marine heat waves and primary production in the New Zealand region. New Zealand Journal of Marine and Freshwater Research, 54(4), 614–635. 10.1080/00288330.2020.1713181

[ece39770-bib-0025] Chiswell, S. M. , Zeldis, J. R. , Hadfield, M. G. , & Pinkerton, M. H. (2017). Wind‐driven upwelling and surface chlorophyll blooms in greater Cook Strait. New Zealand Journal of Marine and Freshwater Research, 51(4), 465–489. 10.1080/00288330.2016.1260606

[ece39770-bib-0026] Clay, Z. , Smith, C. L. , & Blumstein, D. T. (2012). Food‐associated vocalizations in mammals and birds: What do these calls really mean? Animal Behaviour, 83(2), 323–330. 10.1016/j.anbehav.2011.12.008

[ece39770-bib-0027] Collins, M. D. (1993). A split‐step Pade solution for the parabolic equation method. Journal of the Acoustical Society of America, 93(4), 1736–1742. 10.1121/1.406739 35232107

[ece39770-bib-0028] Cotte, C. , d'Ovidio, F. , Chaigneau, A. , Lèvy, M. , Taupier‐Letage, I. , Mate, B. , & Guinet, C. (2011). Scale‐dependent interactions of Mediterranean whales with marine dynamics. Limnology and Oceanography, 56(1), 219–232. 10.4319/lo.2011.56.1.0219

[ece39770-bib-0029] Croll, D. A. , Tershy, B. R. , Hewitt, R. P. , Demer, D. A. , Fiedler, P. C. , Smith, S. E. , Armstrong, W. , Popp, J. M. , Kiekhefer, T. , López, V. R. , Urbán, J. , & Gendron, D. (1998). An integrated approach to the foraging ecology of marine birds and mammals. Deep Sea Research, Part II, 45(7), 1353–1371.

[ece39770-bib-0030] Dall, S. R. X. , Giraldeau, L. A. , Olsson, O. , McNamara, J. M. , & Stephens, D. W. (2005). Information and its use by animals in evolutionary ecology. Trends in Ecology & Evolution, 20(4), 187–193. 10.1016/j.tree.2005.01.010 16701367

[ece39770-bib-0031] Elith, J. , Leathwick, J. R. , & Hastie, T. (2008). A working guide to boosted regression trees. The Journal of Animal Ecology, 77, 802–813. 10.1111/j.1365-2656.2008.01390.x 18397250

[ece39770-bib-0032] Evans, R. , Lea, M. A. , Hindell, M. A. , & Swadling, K. M. (2020). Significant shifts in coastal zooplankton populations through the 2015/16 Tasman Sea marine heatwave. Estuarine, Coastal and Shelf Science, 235, 106538. 10.1016/j.ecss.2019.106538

[ece39770-bib-0033] Frölicher, T. L. , Fischer, E. M. , & Gruber, N. (2018). Marine heatwaves under global warming. Nature, 560, 360–364. 10.1038/s41586-018-0383-9 30111788

[ece39770-bib-0034] Gavrilchuk, K. , Lesage, V. , Fortune, S. M. E. , Trites, A. W. , & Plourde, S. (2021). Foraging habitat of North Atlantic right whales has declined in the Gulf of St. Lawrence, Canada, and may be insufficient for successful reproduction. Endangered Species Research, 44, 113–136. 10.3354/ESR01097

[ece39770-bib-0035] Gavrilov, A. N. , McCauley, R. D. , Salgado‐Kent, C. , Tripovich, J. , & Burton, C. (2011). Vocal characteristics of pygmy blue whales and their change over time. The Journal of the Acoustical Society of America, 130(6), 3651–3660. 10.1121/1.3651817 22225022

[ece39770-bib-0036] Greenwell, B. , Boehmke, B. , & Cunningham, J. (2019). gbm: Generalized Boosted Regression Models. R package version 2.1.5 . https://cran.r‐project.org/package=gbm

[ece39770-bib-0037] Greenwell, B. M. (2017). Pdp: An R package for constructing partial dependence plots. R Journal, 9, 1–16. 10.32614/rj-2017-016

[ece39770-bib-0038] Hazen, E. L. , Jorgensen, S. , Rykaczewski, R. R. , Bograd, S. J. , Foley, D. G. , Jonsen, I. D. , Shaffer, S. A. , Dunne, J. P. , Costa, D. P. , Crowder, L. B. , & Block, B. A. (2013). Predicted habitat shifts of Pacific top predators in a changing climate. Nature Climate Change, 3, 234–238. 10.1038/nclimate1686

[ece39770-bib-0039] Hijmans, R. J. , Phillips, S. , Leathwick, J. R. , & Elith, J. (2017). dismo: Species Distribution Modeling. R package version 1.1‐4 . https://cran.r‐project.org/package=dismo%0A

[ece39770-bib-0040] Hirshfield, M. F. , & Tinkle, D. W. (1975). Natural selection and the evolution of reproductive effort. Proceedings of the National Academy of Sciences of the United States of America, 72(6), 2227–2231. 10.1073/pnas.72.6.2227 1056027 PMC432730

[ece39770-bib-0041] Hoegh‐Guldberg, O. , & Bruno, J. F. (2010). The impact of climate change on the world's marine ecosystems. Science, 328, 1523–1528. 10.1126/science.1189930 20558709

[ece39770-bib-0042] Holbrook, N. J. , Scannell, H. A. , Sen Gupta, A. , Benthuysen, J. A. , Feng, M. , Oliver, E. C. J. , Alexander, L. V. , Burrows, M. T. , Donat, M. G. , Hobday, A. J. , Moore, P. J. , Perkins‐Kirkpatrick, S. E. , Smale, D. A. , Straub, S. C. , & Wernberg, T. (2019). A global assessment of marine heatwaves and their drivers. Nature Communications, 10, 2624. 10.1038/s41467-019-10206-z PMC6570771 31201309

[ece39770-bib-0043] Hyrenbach, K. D. , Forney, K. A. , & Dayton, P. K. (2000). Marine protected areas and ocean basin management. Aquatic Conservation: Marine and Freshwater Ecosystems, 10(6), 437–458. 10.1002/1099-0755(200011/12)10:6<437::AID-AQC425>3.0.CO;2-Q

[ece39770-bib-0044] Kahru, M. , Jacox, M. G. , & Ohman, M. D. (2018). Decrease in the frequency of oceanic fronts and surface chlorophyll concentration in the California current system during the 2014–2016 Northeast Pacific warm anomalies. Deep‐Sea Research Part I, 140, 4–13. 10.1016/j.dsr.2018.04.007

[ece39770-bib-0045] Lavaniegos, B. E. , Jiménez‐Herrera, M. , & Ambriz‐arreola, I. (2019). Unusually low euphausiid biomass during the warm years of 2014–2016 in the transition zone of the California current. Deep‐Sea Research Part II, 169(104638), 169–170. 10.1016/j.dsr2.2019.104638

[ece39770-bib-0046] Laws, R. M. (1959). The foetal growth of whales with special reference to fin whale, Balaenoptera physalus Linn. Discoveries Reports, 29, 281–308.

[ece39770-bib-0047] Lemos, L. S. , Olsen, A. , Smith, A. , Burnett, J. D. , Chandler, T. E. , Larson, S. , Hunt, K. E. , & Torres, L. G. (2021). Stressed and slim or relaxed and chubby? A simultaneous assessment of gray whale body condition and hormone variability. Marine Mammal Science, 1–11, 801–811. 10.1111/mms.12877

[ece39770-bib-0048] Leroy, E. C. , Royer, J. Y. , Alling, A. , Maslen, B. , & Rogers, T. L. (2021). Multiple pygmy blue whale acoustic populations in the Indian Ocean: Whale song identifies a possible new population. Scientific Reports, 11, 8762. 10.1038/s41598-021-88062-5 33888792 PMC8062560

[ece39770-bib-0049] Leroy, E. C. , Samaran, F. , Stafford, K. M. , Bonnel, J. , & Royer, J. Y. (2018). Broad‐scale study of the seasonal and geographic occurrence of blue and fin whales in the southern Indian Ocean. Endangered Species Research, 37, 289–300. 10.3354/esr00927

[ece39770-bib-0050] Letsheleha, I. S. , Shabangu, F. W. , Farrell, D. , Andrew, R. K. , Philip, L. , & Findlay, K. P. (2022). Year ‐ round acoustic monitoring of Antarctic blue and fin whales in relation to environmental conditions off the west coast of South Africa. Marine Biology, 169(41), 1–17. 10.1007/s00227-022-04026-x

[ece39770-bib-0051] Lewis, L. A. , Calambokidis, J. , Stimpert, A. K. , Fahlbusch, J. , Friedlaender, A. S. , McKenna, M. F. , Mesnick, S. L. , Oleson, E. M. , Southall, B. L. , Szesciorka, A. R. , & Širović, A. (2018). Context‐dependent variability in blue whale acoustic behaviour. Royal Society Open Science, 5, 180241. 10.1098/rsos.180241 30225013 PMC6124089

[ece39770-bib-0052] MacArthur, R. H. , & Pianka, E. R. (1966). On optimal use of a patchy environment. The American Naturalist, 100, 603–609. 10.1086/282454

[ece39770-bib-0053] Mackintosh, N. A. , & Wheeler, J. F. G. (1929). Southern blue and fin whales. Discoveries Reports, 1, 257–540.

[ece39770-bib-0054] McCauley, R. D. , Gavrilov, A. N. , Jolli, C. D. , Ward, R. , & Gill, P. C. (2018). Pygmy blue and Antarctic blue whale presence, distribution and population parameters in southern Australia based on passive acoustics. Deep‐Sea Research Part II, 158, 154–168. 10.1016/j.dsr2.2018.09.006

[ece39770-bib-0055] McDonald, M. A. , Mesnick, S. L. , & Hildebrand, J. A. (2006). Biogeographic characterisation of blue whale song worldwide: Using song to identify populations. Journal of Cetacean Research and Management, 8(1), 55–65. 10.1029/2006WR005124.DOI

[ece39770-bib-0056] McNamara, J. M. , & Houston, A. I. (1986). The common currency for behavioral decisions. The American Naturalist, 127(3), 358–778. 10.1086/284489

[ece39770-bib-0057] Mellinger, D. K. , & Clark, C. W. (2000). Recognizing transient low‐frequency whale sounds by spectrogram correlation. The Journal of the Acoustical Society of America, 107(6), 3518–3529. 10.1121/1.429434 10875396

[ece39770-bib-0058] Mellinger, D. K. , Roch, M. A. , Nosal, E.‐M. , & Klinck, H. (2016). Signal processing. In W. W. L. Au & M. O. Lammers (Eds.), Listening in the ocean (pp. 359–409). Springer.

[ece39770-bib-0059] Mendelssohn, R. (2019). rerddapXtracto: Extracts Environmental Data from “ERDDAP” Web Services . https://github.com/rmendels/rerddapXtracto%0A

[ece39770-bib-0060] Nicol, S. , Pauly, T. , Bindoff, N. L. , Wright, S. , Thiele, D. , Hosle, G. W. , Strutton, P. G. , & Woehler, E. (2000). Ocean circulation off East Antarctica affects ecosystem structure and sea‐ice extent. Nature, 406, 504–507. 10.1038/35020053 10952309

[ece39770-bib-0061] Oestreich, W. K. , Abrahms, B. , Mckenna, M. F. , Goldbogen, J. A. , Crowder, L. B. , & Ryan, J. P. (2022). Acoustic signature reveals blue whales tune life history transitions to oceanographic conditions. Functional Ecology, 00, 1–14. 10.1111/1365-2435.14013

[ece39770-bib-0062] Oestreich, W. K. , Fahlbusch, J. A. , Cade, D. E. , Calambokidis, J. , Margolina, T. , Joseph, J. , Friedlaender, A. S. , McKenna, M. F. , Stimpert, A. K. , Southall, B. L. , Goldbogen, J. A. , & Ryan, J. P. (2020). Animal‐borne metrics enable acoustic detection of blue whale migration. Current Biology, 30, 1–7. 10.1016/j.cub.2020.08.105 33007246

[ece39770-bib-0063] Oleson, E. M. , Calambokidis, J. , Burgess, W. C. , McDonald, M. A. , LeDuc, C. A. , & Hildebrand, J. A. (2007). Behavioral context of call production by eastern North Pacific blue whales. Marine Ecology Progress Series, 330, 269–284. 10.3354/meps330269

[ece39770-bib-0064] Oleson, E. M. , Wiggins, S. M. , & Hildebrand, J. A. (2007). Temporal separation of blue whale call types on a southern California feeding ground. Animal Behaviour, 74, 881–894. 10.1016/j.anbehav.2007.01.022

[ece39770-bib-0065] Oliver, E. C. J. , Benthuysen, J. A. , Bindoff, N. L. , Hobday, A. J. , Holbrook, N. J. , Mundy, C. N. , & Perkins‐Kirkpatrick, S. E. (2017). The unprecedented 2015/16 Tasman Sea marine heatwave. Nature Communications, 8, 16101. 10.1038/ncomms16101 PMC5519980 28706247

[ece39770-bib-0066] Oliver, E. C. J. , Donat, M. G. , Burrows, M. T. , Moore, P. J. , Smale, D. A. , Alexander, L. V. , Benthuysen, J. A. , Feng, M. , Gupta, A. S. , Hobday, A. J. , Holbrook, N. J. , Perkins‐Kirkpatrick, S. E. , Scannell, H. A. , Straub, S. C. , & Wernberg, T. (2018). Longer and more frequent marine heatwaves over the past century. Nature Communications, 9(1324), 1–12. 10.1038/s41467-018-03732-9 PMC5893591 29636482

[ece39770-bib-0067] Perryman, W. L. , Joyce, T. , Weller, D. W. , & Durban, J. W. (2021). Environmental factors influencing eastern North Pacific gray whale calf production 1994–2016. Marine Mammal Science, 37(2), 448–462. 10.1111/mms.12755

[ece39770-bib-0068] Piatt, J. F. , Parrish, J. K. , Renner, H. M. , Schoen, S. K. , Jones, T. T. , Arimitsu, M. L. , Kuletz, K. J. , Bodenstein, B. , García‐Reyes, M. , Duerr, R. S. , Corcoran, R. M. , Kaler, R. S. A. , McChesney, G. J. , Golightly, R. T. , Coletti, H. A. , Suryan, R. M. , Burgess, H. K. , Lindsey, J. , Lindquist, K. , … Sydeman, W. J. (2020). Extreme mortality and reproductive failure of common murres resulting from the Northeast Pacific marine heatwave of 2014–2016. PLoS One, 15(1), e0226087. 10.1371/journal.pone.0226087 31940310 PMC6961838

[ece39770-bib-0069] Pirotta, E. , Mangel, M. , Costa, D. P. , Goldbogen, J. , Harwood, J. , Hin, V. , Irvine, L. M. , Mate, B. R. , McHuron, E. A. , Palacios, D. M. , Schwarz, L. K. , & New, L. (2019). Anthropogenic disturbance in a changing environment: Modelling lifetime reproductive success to predict the consequences of multiple stressors on a migratory population. Oikos, 128(9), 1340–1357. 10.1111/oik.06146

[ece39770-bib-0070] Poloczanska, E. S. , Brown, C. J. , Sydeman, W. J. , Kiessling, W. , Schoeman, D. S. , Moore, P. J. , Brander, K. , Bruno, J. F. , Buckley, L. B. , Burrows, M. T. , Duarte, C. M. , Halpern, B. S. , Holding, J. , Kappel, C. V. , O'Connor, M. I. , Pandolfi, J. M. , Parmesan, C. , Schwing, F. , Thompson, S. A. , & Richardson, A. J. (2013). Global imprint of climate change on marine life. Nature Climate Change, 3(919–925), 919–925. 10.1038/nclimate1958

[ece39770-bib-0071] Samaran, F. , Guinet, C. , Adam, O. , Motsch, J.‐F. , & Cansi, Y. (2010). Source level estimation of two blue whale subspecies in southwestern Indian Ocean. The Journal of the Acoustical Society of America, 127(6), 3800–3808. 10.1121/1.3409479 20550278

[ece39770-bib-0072] Samaran, F. , Stafford, K. M. , Branch, T. A. , Gedamke, J. , Royer, J. , Dziak, R. P. , & Guinet, C. (2013). Seasonal and geographic variation of southern blue whale subspecies in the Indian Ocean. PLoS One, 8(8), e71561. 10.1371/journal.pone.0071561 23967221 PMC3742792

[ece39770-bib-0073] Santora, J. A. , Mantua, N. J. , Schroeder, I. D. , Field, J. C. , Hazen, E. L. , Bograd, S. J. , Sydeman, W. J. , Wells, B. K. , Calambokidis, J. , Saez, L. , Lawson, D. , & Forney, K. A. (2020). Habitat compression and ecosystem shifts as potential links between marine heatwave and record whale entanglements. Nature Communications, 11(536), 1–12. 10.1038/s41467-019-14215-w PMC6985238 31988285

[ece39770-bib-0074] Schall, E. , Di Iorio, L. , Berchok, C. , Filún, D. , Bedriñana‐Romano, L. , Buchan, S. J. , Van Opzeeland, I. , Sears, R. , & Hucke‐Gaete, R. (2019). Visual and passive acoustic observations of blue whale trios from two distinct populations. Marine Mammal Science, 1–10, 365–374. 10.1111/mms.12643

[ece39770-bib-0075] Seyboth, E. , Groch, K. R. , Dalla Rosa, L. , Reid, K. , Flores, P. A. C. , & Secchi, E. R. (2016). Southern right whale (*Eubalaena australis*) reproductive success is influenced by krill (*Euphausia superba*) density and climate. Scientific Reports, 6, 28205. 10.1038/srep28205 27306583 PMC4910057

[ece39770-bib-0076] Shirtcliffe, T. G. L. , Moore, M. I. , Cole, A. G. , Viner, A. B. , Baldwin, R. , & Chapman, B. (1990). Dynamics of the cape farewell upwelling plume, New Zealand. New Zealand Journal of Marine and Freshwater Research, 24(4), 555–568. 10.1080/00288330.1990.9516446

[ece39770-bib-0077] Silber, G. K. , Lettrich, M. D. , Thomas, P. O. , Baker, J. D. , Baumgartner, M. , Becker, E. A. , Boveng, P. , Dick, D. M. , Fiechter, J. , Forcada, J. , Forney, K. A. , Griffis, R. B. , Hare, J. A. , Hobday, A. J. , Howell, D. , Laidre, K. L. , Mantua, N. , Quakenbush, L. , Santora, J. A. , … Waples, R. S. (2017). Projecting marine mammal distribution in a changing climate. Frontiers in Marine Science, 4, 413. 10.3389/fmars.2017.00413

[ece39770-bib-0078] Širović, A. , Hildebrand, J. A. , Wiggins, S. M. , & Thiele, D. (2009). Blue and fin whale acoustic presence around Antarctica during 2003 and 2004. Marine Mammal Science, 25(1), 125–136. 10.1111/j.1748-7692.2008.00239.x

[ece39770-bib-0079] Soledade Lemos, L. , Burnett, J. D. , Chandler, T. E. , Sumich, J. L. , & Torres, L. G. (2020). Intra‐ and inter‐annual variation in gray whale body condition on a foraging ground. Ecosphere, 11(4), e03094. 10.1002/ecs2.3094

[ece39770-bib-0080] Stafford, K. M. , Bohnenstiehl, D. R. , Tolstoy, M. , Chapp, E. , Mellinger, D. K. , & Moore, S. E. (2004). Antarctic‐type blue whale calls recorded at low latitudes in the Indian and eastern Pacific oceans. Deep Sea Research Part I: Oceanographic Research Papers, 51, 1337–1346. 10.1016/j.dsr.2004.05.007

[ece39770-bib-0081] Stafford, K. M. , Nieukirk, S. L. , & Fox, C. G. (2001). Geographic and seasonal variation of blue whale calls in the North Pacific. Journal of Cetacean Reasearch and Management, 3(1), 65–76.

[ece39770-bib-0082] Sutton, P. J. H. , & Bowen, M. (2019). Ocean temperature change around New Zealand over the last 36 years. New Zealand Journal of Marine and Freshwater Research, 53, 305–326. 10.1080/00288330.2018.1562945

[ece39770-bib-0083] Sydeman, W. J. , Poloczanska, E. , Reed, T. E. , & Thompson, S. A. (2015). Climate change and marine vertebrates. Science, 350, 772–777. 10.1126/science.aac9874 26564847

[ece39770-bib-0084] Szesciorka, A. R. , Ballance, L. T. , Širovic, A. , Rice, A. , Ohman, M. D. , Hildebrand, J. A. , & Franks, P. J. S. (2020). Timing is everything: Drivers of interannual variability in blue whale migration. Scientific Reports, 10(7710), 1–9. 10.1038/s41598-020-64855-y 32382054 PMC7206123

[ece39770-bib-0085] Torney, C. J. , Berdahl, A. , & Couzin, I. D. (2011). Signalling and the evolution of cooperative foraging in dynamic environments. PLoS Computational Biology, 7(9), e1002194. 10.1371/journal.pcbi.1002194 21966265 PMC3178622

[ece39770-bib-0086] Torres, L. G. (2013). Evidence for an unrecognised blue whale foraging ground in New Zealand. New Zealand Journal of Marine and Freshwater Research, 47(2), 235–248. 10.1080/00288330.2013.773919

[ece39770-bib-0087] Torres, L. G. , Barlow, D. R. , Chandler, T. E. , & Burnett, J. D. (2020). Insight into the kinematics of blue whale surface foraging through drone observations and prey data. PeerJ, 8, e8906. 10.7717/peerj.8906 32351781 PMC7183305

[ece39770-bib-0088] Tyack, P. L. , & Clark, C. W. (2000). Communication and acoustic behavior of dolphins and whales. In Hearing by whales and dolphins (pp. 156–224). Springer. 10.1007/978-1-4612-1150-1_4

[ece39770-bib-0089] Warren, V. E. , Širović, A. , McPherson, C. , Goetz, K. T. , Radford, C. A. , & Constantine, R. (2021). Passive acoustic monitoring reveals Spatio‐temporal distributions of Antarctic and pygmy blue whales around Central New Zealand. Frontiers in Marine Science, 7(January), 1–14. 10.3389/fmars.2020.575257

[ece39770-bib-0090] Wentworth, C. K. (1922). A scale of grade and class terms for clastic sediments. The Journal of Geology, 30(5), 377–392. 10.1086/622910

[ece39770-bib-0093] Wright, M. N. , & Ziegler, A. (2017). ranger: A fast implementation of random forests for high dimensional data in C++ and R. Journal of Statistical Software, 77(1). 10.18637/jss.v077.i01

[ece39770-bib-0091] Yablokov, A. V. (1994). Validity of whaling data. Nature, 108(376), 108.

[ece39770-bib-0092] Zemsky, V. A. , Berzin, A. , Mikhalyev, Y. A. , & Tormosov, D. D. (1995). *Soviet Antarctic whaling data* (*1947–1972*).

